# Sodium oligomannate disrupts the adherence of Rib^high^ bacteria to gut epithelia to block SAA-triggered Th1 inflammation in 5XFAD transgenic mice

**DOI:** 10.1038/s41421-024-00725-5

**Published:** 2024-11-19

**Authors:** Xinyi Wang, Zuoquan Xie, Jie Yuan, Enjing Jin, Wen Lian, Shuaishuai Chang, Guangqiang Sun, Zhengnan Feng, Hui Xu, Chen Du, Xinying Yang, Aihua Xia, Ji Qiu, Qingli Zhang, Feifei Lin, Jia Liu, Liang Li, Xiaoguang Du, Zhongping Xiao, Zhou Yi, Zhiyu Luo, Changrong Ge, Rui Li, Mingyue Zheng, Yi Jiang, Tao Wang, Jing Zhang, Qihao Guo, Meiyu Geng

**Affiliations:** 1 State Key Laboratory of Drug Research, Shanghai Institute of Materia Medica, Chinese Academy of Sciences, Shanghai, China; 2 Shanghai Green Valley Pharmaceutical Co. Ltd, Shanghai, China; 3 Institutional Technology Service Centre, Shanghai Institute of Materia Medica, Chinese Academy of Sciences, Shanghai, China; 4 School of Pharmacy, China Pharmaceutical University, Nanjing, Jiangsu China; 5 Drug Discovery and Design Center, State Key Laboratory of Drug Research, Shanghai Institute of Materia Medica, Chinese Academy of Sciences, Shanghai, China; 6 University of Chinese Academy of Sciences, Beijing, China; 7 Lingang Laboratory, Shanghai, China; 8 Department of Psychiatry and Affective Disorder Center, Ruijin Hospital Affiliated to Shanghai Jiao Tong University, School of Medicine, Shanghai, China; 9 Department of Gerontology, Shanghai Jiao Tong University Affiliated Sixth People’s Hospital, Shanghai, China; 10 Shandong Laboratory of Yantai Drug Discovery, Bohai Rim Advanced Research Institute for Drug Discovery, Yantai, Shandong China

**Keywords:** Molecular biology, Ageing, Immunology

## Abstract

Sodium oligomannate (GV-971), an oligosaccharide drug approved in China for treating mild-to-moderate Alzheimer’s disease (AD), was previously found to recondition the gut microbiota and limit altered peripheral Th1 immunity in AD transgenic mice. As a follow-up study, we here made advances by pinpointing a *Lactobacillus murinus* (*L.m*.) strain that highly expressed a gene encoding a putative adhesin containing Rib repeats (Rib^high^-*L.m*.) particularly enriched in 5XFAD transgenic mice. Mechanistically, Rib^high^-*L.m*. adherence to the gut epithelia upregulated fecal metabolites, among which lactate ranked as the top candidate. Excess lactate stimulated the epithelial production of serum amyloid A (SAA) in the gut via the GPR81-NFκB axis, contributing to peripheral Th1 activation. Moreover, GV-971 disrupted the adherence of Rib^high^-*L.m*. to gut epithelia via direct binding to Rib, which corrected the excess lactate, reduced SAA, and alleviated Th1-skewed inflammation. Together, we gained further insights into the molecular link between gut bacteria and AD progression and the mechanism of GV-971 in treating AD.

## Introduction

Gut microbiota dysbiosis is closely related to the progression of Alzheimer’s disease (AD). Intervention of gut microbiota diversity and composition has been found to reduce deposition of amyloid beta (Aβ) plaque and alter plaque-localized microglia morphology and activation status, raising interest in investigating the roles of microbiome in AD pathogenesis^1,2^. Lately, the remarkable advancement in multi-omics sequencing of microbiology has revolutionized the field^3–5^, leading to the identification of AD-associated gut microbiota change across genus and species levels. Meanwhile, mechanistic links that illustrate how microorganism influences the brain are increasingly appreciated^6^. Microbiome-associated metabolic, immunological, and neurochemical factors all could ultimately impact the nervous system^6^. For example, the alteration of butyrate-producing bacteria has been linked to T-cell imbalance, epithelial barrier leakage (“leaky gut”), and the increased bacterial translocation in AD^7^. Various bacterial metabolites, including short chain fatty acids, kynurenine, and amino acids, have been revealed as neurotransmitter precursors or energy sources for immune cells implicated in AD development^7–11^.

Despite the advancement, the intrinsic connection between gut imbalances and AD remains mostly obscure, due to the complexity of AD pathogenesis and disease’s subtle and evolving nature. In fact, distinct stages of AD display variable gut microbiota imbalances, leading to stage-specific shifts of microbiome composition as the disease develops^7,12^. Therefore, a precise illustration of the crosstalk between gut microbiota and the host during different AD stages, together with stage-specific metabolite changes and peripheral inflammatory status, is crucial for understanding the cause-and-effect mechanisms underlying gut dysbiosis in AD pathogenesis.

In a previous study using the 5XFAD transgenic (Tg) mouse model, we discovered that sodium oligomannate (GV-971), an anti-AD oligosaccharide drug approved for clinical use for mild-to-moderate AD patients, corrects gut microbiota imbalances and associated phenylalanine and isoleucine level changes. These effects are associated with reduced Th1 cell infiltration into the brain, decreased neuroinflammation and Aβ deposition, and enhanced cognitive performance^13^. More recently, an independent study also validated the modulatory effect of GV-971 on gut microbiota to reduce cerebral amyloidosis and reactive microglia^14^. Despite the therapeutic promise and mechanistic advancement, the underlying mechanism of GV-971 is not well understood. Specifically, it remains unclear how GV-971 remodels the gut microbiome, which bacterial species essentially contribute to its therapeutic benefit, and how bacterial metabolites trigger Th1 activation. Moreover, the aforementioned findings were mainly observed in mice models up to 36 weeks of age^15^. It will be interesting to illustrate how these changes manifest in ages of more than 36 weeks and whether different stages exhibit different profiles.

In this study, we used the 5XFAD Tg mice model up to 41 weeks of age to gain in-depth insights into molecular links between the gut microbiome alteration and specific Th1 cell activation that are implicated in AD progression. Taking advantage of metagenomics that empowered an in-depth profiling of bacterial changes down to the species level, we were able to pinpoint a single bacteria strain, *Lactobacillus murinus* (*L.m*.) featured with high expression of adhesin containing Rib repeats (Rib^high^-*L.m*.) that was closely associated with AD progression. The identification of this strain allowed us to discover bacterial metabolite changes, which not only echoed our previous findings regarding phenylalanine and isoleucine, but also highlighted the critical roles of excessive lactate functioning as signal molecule to activate the epithelial production of serum amyloid A (SAA) to drive the systemic inflammation. Importantly, the current study also revealed the direct binding of GV-971 to Rib repeats of Rib^high^-*L.m*. This specific binding disrupted the adherence of Rib^high^-*L.m.* to gut epithelia and as a result, reduced the epithelial production of SAA and alleviated SAA-promoted Th1 inflammation.

## Results

### Alterations in gut microbiota composition and metabolic activity are associated with gut inflammatory milieu in 5XFAD transgenic mice

**Fig. 1:**
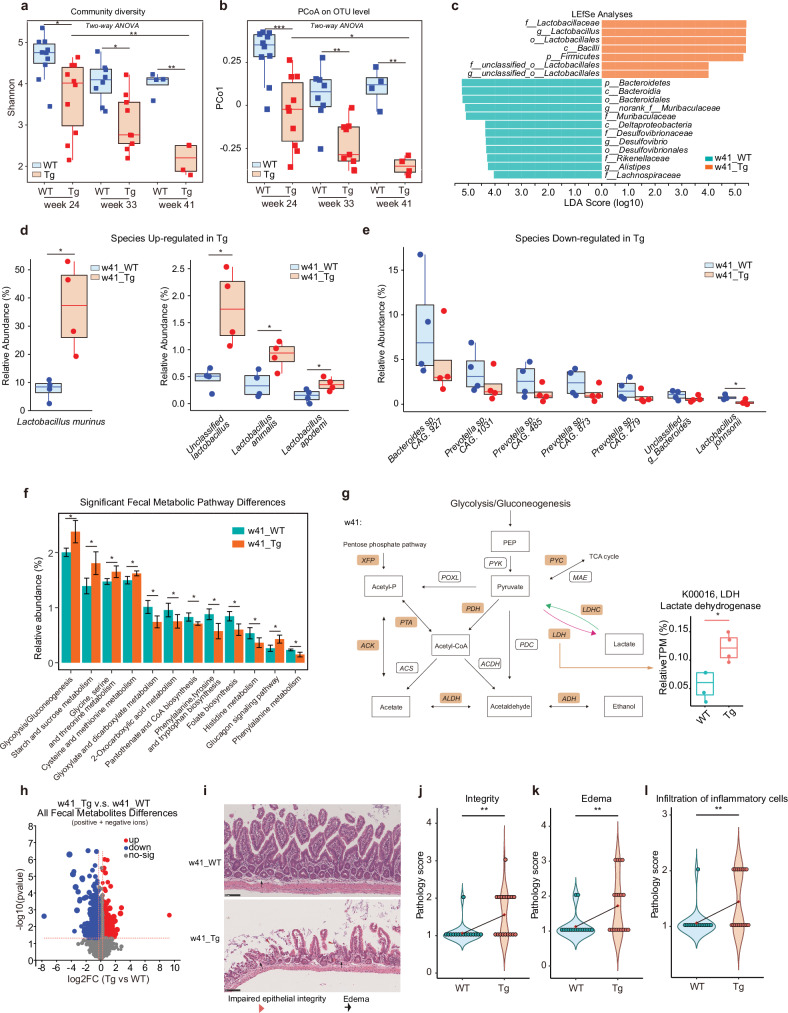
Alteration of gut microbiota diversity and abundance, metabolic activity, and gut inflammatory milieu in 5XFAD Tg mice from 24, 33 to 42 weeks of age. **a** Shannon diversity of the gut fecal microbiota of WT and 5XFAD Tg mice at week ages 24, 33, and 41 sequenced by 16S rRNA-seq. Statistical significance was determined using two-way ANOVA. Effects of Time and Genotype were significant (Time: *P* < 0.001; Genotype: *P* < 0.001). Post-hoc analysis for single experimental conditions using Tukey’s Honest Significant Difference (HSD) test: w24_WT vs w24_Tg: *, *P*_adj_ = 0.0201389; w33_WT vs w33_Tg: *, *P*_adj_ = 0.0319952; w41_WT vs w41_Tg: **, *P*_adj_ = 0.0028909; w24_Tg vs w41_Tg: **, *P*_adj_ = 0.0037371. Blue, WT; red, Tg. **b** PCoA on Operational Taxonomy Units (OTU) level of gut fecal microbiota of WT and Tg mice at week ages 24, 33 and 41 (*n* = 4–10) sequenced by 16S rRNA-seq. PCo1, principal coordinate 1. Statistical significance was determined using two-way ANOVA. Effects of Time and Genotype were significant (Time: *P* < 0.001; Genotype: *P* < 0.001). Post-hoc analysis for single experimental conditions using Tukey’s HSD test: w24_WT vs w24_Tg: ***, *P*_adj_ = 0.0002200; w33_WT vs w33_Tg: **, *P*_adj_ = 0.0060697; w41_WT vs w41_Tg: **, *P*_adj_ = 0.0021552; w24_Tg vs w41_Tg: *, *P*_adj_ = 0.0195302. Blue, WT; red, Tg. **c** Linear discriminant analysis effect size (LEfSe) and linear discriminant analysis (LDA) based on OTU were used to differentiate key bacteria between the fecal microbiota of WT and Tg mice at week age 41 (w41) from phylum to genus level sequenced by 16S rRNA-seq. The log_10_LDA cut-off score was set to 4.0 to indicate representative microbiota of each group that had significant differential power between WT and Tg mice at w41. p, phylum; c, class; o, order; f, family; g, genus. Green, WT; red, Tg. **d**, **e** Changes of the relative abundance of the gut fecal microbiota of WT and Tg mice that are significantly higher (**d**) or largely lower (**e**) in Tg relative to WT at w41 at species level, revealed by metagenomics. Relative abundance is calculated based on total reads per million (TPM). *n* = 4. *P < 0.05, two-group comparison by Student’s *t*-test. Blue, WT; red, Tg. **f** Changes of the relative abundance of functional pathways enriched in the gut fecal microbiota of WT and Tg mice at w41, revealed by metagenomics. Relative abundance is calculated based on TPM. Data are represented as mean ± standard deviation (SD) (*n* = 4). *P < 0.05, two-group comparison by Student’s *t*-test. Green, WT; red, Tg. **g** Mean relative abundance changes of several key enzymes of microbial pathways related to glycolysis and lactate production of WT and Tg mice feces at w41. The relative abundances of XFP (*P* < 0.05) and ACK (*P* < 0.05) and LDH (*P* < 0.05) were significantly more abundant in the gut microbiota of the w41 Tg mice feces (*n* = 4 for each group). Yellow-filled brackets: enzymes that up-regulated in w41 Tg gut feces compared to WT feces. ACDH, Acetaldehyde dehydrogenase; ACK, Acetate kinase; ACS, Acetyl-CoA synthetase; ADH, Alcohol dehydrogenase; ALDH, Aldehyde dehydrogenase; LDHC, Lactate dehydrogenase (cytochrome); LDH, Lactate dehydrogenase; MAE, Malate dehydrogenase; PDC, Pyruvate decarboxylase; PDH, Pyruvate dehydrogenase E1; POXL, Pyruvate oxidase; PTA, Phosphate acetyltransferase; PYC, Pyruvate carboxylase; PYK, Pyruvate kinase; XFP, Xylulose-5-p/f-6-p phosphoketolase. TCA cycle, tricarboxylic acid cycle. *P < 0.05, by Student’s *t*-test. **h** Volcano plot shows differential detected metabolites in the gut fecal microbiota of WT and Tg mice at w41 using untargeted metabolomics. Red dots: up-regulated in Tg mice feces; blue dots: down-regulated in Tg mice feces; gray dots: no-sig, not significantly changing metabolites. The significance level is defined as a *P* value less than or equal to 0.05 and the absolute value of Fold Change (FC) of metabolites abundances in Tg feces vs WT feces value greater than 1.2 or smaller than 0.83. **i** Representative HE staining histology sections from the ileum tissue of WT and Tg mice at w41. Red triangle, epithelial lesions reflecting impaired epithelial integrity status; black arrow, epithelial edema. The scale bar is 100 μm. WT: *n* = 5, 5 M; Tg: *n* = 6, 4 M + 2 F. M, male mice; F, female mice. **j**–**l** Statistics of pathology evaluation scores on gut integrity (**j**), edema (**k**), and infiltration of inflammatory cells (**l**) of ileum tissue of WT and Tg mice at w41. Higher pathology scores represent less integrity, more severe edema, and more infiltration of inflammatory cells, respectively. Blue, WT; red, Tg. The small red diamond symbol at the center of the violin plot is the mean value of all score points of each specific group. **P < 0.01, two-group comparison by Student’s *t*-test. Box plots represent the median (the horizontal line within boxes) and the 75th and 25th percentiles (the top and bottom of each box, respectively). The upper and lower whiskers represent 1.5× IQR from the top and bottom of the box, respectively. w24_WT: *n* = 10, 5 M + 5 F; w24_Tg: *n* = 10, 5 M + 5 F; w33_WT: *n* = 8, 4 M + 4 F; w33_Tg: *n* = 9, 5 M + 4 F; w41_WT: *n* = 4, 3 M + 1 F; w41_Tg: *n* = 4, 4 F. M, male mice; F, female mice.

Our previous study revealed AD-associated microbiota changes using 24 to 36-week-old Tg mice. To better reflect AD progression during the aging process in AD progression, we used Tg mice up to 41-week age and the age-matched wild-type (WT) mice as reference controls. 16S rRNA sequencing (rRNA-seq) of the fecal samples revealed a significant decrease in gut microbiota diversity and community richness in Tg mice along with disease progression from 24 to 41 weeks of age, as evidenced by various indexes (Fig. 1a; Supplementary Fig. S1a, b). Of note, the most pronounced differences between WT and Tg mice occurred at 41 weeks of age, the period of which was not covered in our previous study^13^. The principal coordinate analysis (PCoA) indicated marked distinctions in the microbiota composition of WT and Tg mice as they aged, revealed by the PCo1 value (Fig. 1b), aligning well with our prior study^13^.

To discern the shifts in microbiome composition during AD progression, we investigated variations in the relative abundance of bacteria. Notably, at both phylum and family levels, our results mirrored our earlier findings^13^, showing a similar trend lasting up to 41 weeks of age (Supplementary Fig. S1c, d). Specifically, in Tg mice, we noted a significant increase in the *Firmicutes* phylum and the *Lactobacillaceae* family, coupled with a decrease in the *Bacteroidetes* phylum and the *Muribaculaceae* family (Supplementary Fig. S1c, d). Importantly, we here discovered a significantly increased proportion of anaerobic and gram-positive bacteria, consistent with the phenotype of *Lactobacillus* genus^16^ that were not noted in our previous study (Supplementary Fig. S1e, f)^13^. At 41 weeks of age, bacteria belonging to the *Lactobacillaceae* family were particularly enriched in Tg mice compared to WT mice. Conversely, *Bacteroidia* and *Muribaculaceae* were more prevalent in WT mice than in Tg mice (Fig. 1c).

To further pinpoint changes at the species level, we employed metagenomic sequencing. We paid special attention to bacterial alterations observed at 41 weeks of age, which led to the identification of a significant increase in the relative abundance of *L.m*. in Tg mice compared to WT mice. Other species within the *Lactobacillus* genus like *Lactobacillus animalis*, etc., showed similar trends (Fig. 1d). Conversely, 41-week-old Tg mice exhibited lower abundances of *Bacteroidetes sp. CAG.927*, *Prevotella sp. CAG.1031*, and so on (Fig. 1e). To confirm these findings, we took the approaches of fecal microbiota transplantation (FMT) and antibiotics (ABX) treatment. Transplanting Tg feces into the recipient Tg mice produced similar bacteria changes as observed in Tg mice, i.e., increased abundance of *L.m*. and the decreased abundance of some *Bacteroides* and *Prevotella* species (Supplementary Fig. S1g, upper panel). By contrast, when Tg mice underwent ABX treatment, the abundance of *L.m*. decreased, and there was an increase in the abundance of *unclassified_g_Bacteroides* species in 41-week-old Tg mice (Supplementary Fig. S1g, lower panel). These findings suggested that the increased *L.m*. and the reduced *Bacteroides* and *Prevotella* species were associated with AD.

As the metabolic activity of the gut microbiota is crucial for its interaction with the host, we analyzed alterations in gut microbiota metabolic pathways using metagenomics^17^. Various metabolic pathways were substantially altered in Tg mice at 41 weeks of age compared to the age-matched WT mice, including phenylalanine-related pathways identified in our previous findings using 36-week-old Tg mice^13^ (Fig. 1f). In fact, the significant deregulation in glycolysis/gluconeogenesis, fatty acid pathways, and phenylalanine metabolism pathways between Tg vs WT mice lasted from 24 to 41 weeks of age (Supplementary Fig. S2a). Furthermore, we observed significantly upregulated lactate dehydrogenase (LDH), an enzyme catalyzing the lactate production in Tg (Fig. 1g). Importantly, these alterations in metabolic pathways were linked to the known functions of the altered bacterial species, i.e., the increase in glycolysis/gluconeogenesis was closely associated with increased *Lactobacillus*^18^, while the decrease in phenylalanine metabolism was likely associated with the decreased presence of *Bacteroidetes*^19,20^.

To further validate the observed genomic changes, we employed untargeted metabolomics to assess the alteration of fecal metabolites. Principal component analysis (PCA) of the detected metabolites revealed a distinct pattern in the feces of 41-week-old Tg mice compared to that of WT mice (Supplementary Fig. S2b, c). We showed that the volcano plot of all metabolites exhibited clear differences in the gut microbiota metabolite profiles between Tg mice and WT mice at 41 weeks of age (Fig. 1h), indicating that Tg mice at 41 weeks experienced significant metabolic reprogramming.

As gut metabolite alteration has been linked to a defect in mucosal integrity and immune balance^19,21^, we then asked whether the gut epithelium might be exposed to a proinflammatory environment due to metabolic disorders. Hematoxylin-eosin (HE) staining of ileum tissue sections from both WT and Tg mice at 41 weeks of age revealed a notable deterioration in epithelial integrity within the gastrointestinal tract in Tg mice (Fig. 1i, j), as marked by structural irregularities in the gut lining. The structural changes were particularly associated with increased intestinal edema (Fig. 1k), as indicated by the presence of tissue swelling and heightened inflammation in the gut of Tg mice, which were absent in that of WT mice. Notably, immune cell infiltration was also significantly elevated in the affected areas (Fig. 1l), suggesting an ongoing immune response that might potentially signify a proinflammatory milieu within the gut epithelium of Tg mice.

### Altered gut microbiota induces gut epithelial SAA to activate peripheral innate immune response and Th1-skewed inflammation

**Fig. 2:**
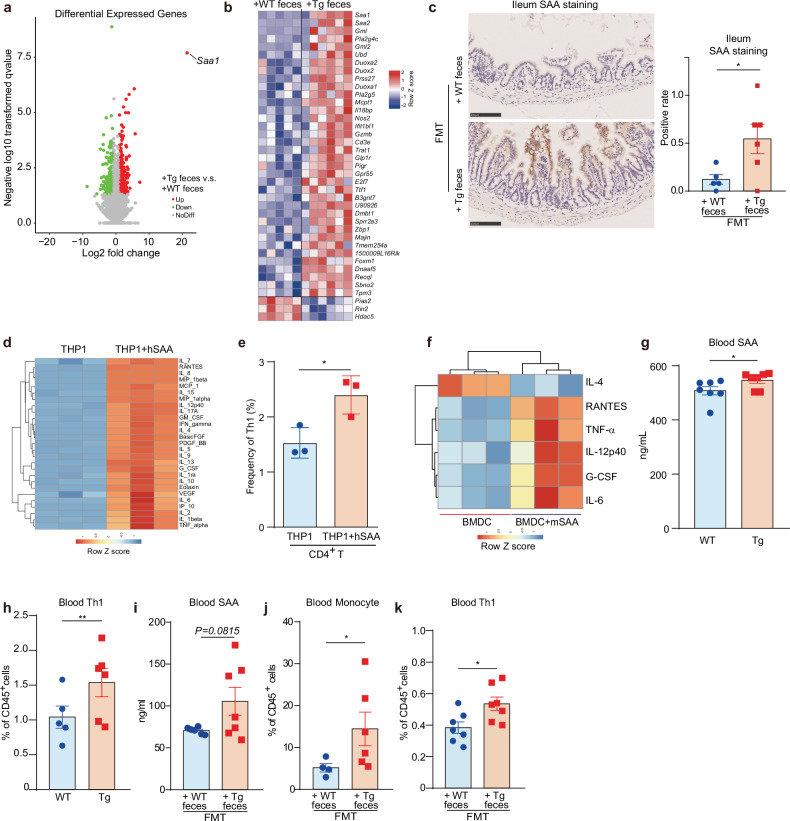
Deregulated ileum epithelial SAA activates peripheral innate immune response and Th1-skewed inflammation. **a** Volcano plot shows differentially expressed genes (DEGs) of the ileum tissue of 7-month-old Tg recipient mice that received 41-week-age WT (+WT feces) and 5XFAD (Tg) feces (+Tg feces) in FMT assays. Up, down: significantly expressed genes up-regulated and down-regulated respectively in ileum tissue of 7-month-old Tg recipient mice that received 41-week-age Tg feces (+ Tg feces) vs WT feces (+ WT feces). NoDiff, not differentially expressed genes. The significance level is defined as *P*_adj_ < 0.05 and the absolute value of log_2_FC of +Tg feces vs +WT feces > 1. **b** Heatmap shows the differential gene expression of the ileum tissue. The cut-off *P* value is 0.05, while the cut-off *P*_adj_ value is 0.3. Red or blue colors indicate up- or down-regulation in the ileum tissue of Tg mice that received Tg fecal samples compared to those receving WT feces, respectively. **c** Representative IHC images stained with SAA antibody of the ileum tissue slices of 7-month-old Tg recipient mice that received 41-week-age WT (+WT feces) and Tg feces (+Tg feces) in FMT assays. Data are represented as mean ± standard error of the mean (SEM). The yellow staining area is the positive SAA staining signal. The positive rate is calculated as the ratio of positive area × positive staining intensity. *P < 0.05, by Student’s *t-*test, *n* = 5–6. **d** Heatmap shows the changes of cytokines secreted by THP1-Dual cells stimulated by hSAA. THP1-Dual cells were treated with 0.8 µg/mL hSAA for 24 h, and the secretion of cytokines in the culture supernatant was determined by cytokine array. *n* = 3. **e** Vehicle-induced or hSAA-induced (10 µg/mL, 24 h) THP1-Dual cells were co-cultured with naïve CD4^+^ T cells for 3 days, and the frequency of Th1 cells was detected by the flow cytometer. Data are mean ± SEM. *P < 0.05, by Student’s *t*-test, *n* = 3. **f** BMDCs were incubated with 10 µg/mL mSAA for 24 h, and the secretion of cytokines in the culture supernatant was determined by cytokine array, and the significantly changed cytokines in mSAA-treated group vs control group were shown as heatmap. *n* = 3. **g** Level of the blood SAA of WT and Tg mice at w41 measured by enzyme-linked immune absorbent (ELISA) assays. Data are represented as mean ± SEM. *P < 0.05 by Student’s *t*-test, *n* = 7. WT: *n* = 7, 3 M + 4 F; Tg: *n* = 7, 3 M + 4 F. M, male mice; F, female mice. **h** Changes in the frequency of blood Th1 cells of WT and Tg mice at w 41 measured by flow cytometry. Data are represented as mean ± SEM, **P < 0.01 by Student’s *t*-test, *n* = 5–6. WT: *n* = 5, 5 M; Tg: *n* = 6, 4 M + 2 F. M, male mice; F, female mice. **i** Mouse blood SAA levels of the 12-month-old C57 WT recipient mice that received 41-week-age WT or Tg fecal samples in FMT assays. Data are represented as mean ± SEM. *n* = 6–7. **j** Cell frequency of the blood monocyte of the recipient 7-month-old Tg mice that received 41-week-age WT or Tg feces in FMT assay. Data are represented as mean ± SEM. *P < 0.05 by Student’s *t*-test, *n* = 4–6. **k** Frequency of blood Th1 cells of the 12-month-old C57 WT recipient mice that received 41-week-age WT (+ WT feces) or Tg fecal samples (+ Tg feces) in FMT assays. Data are represented as mean ± SEM. *P < 0.05, by Student’s *t*-test, *n* = 7. All recipient mice in FMT assays are male mice.

To establish causal links between the aforementioned associations, we focused on the terminal ileum, a pivotal interface connecting host immune responses with bacteria alterations^22–24^. We conducted RNA-sequencing (RNA-seq) of ileum tissues freshly dissected from mice that received fecal transplants from 41-week-old Tg or WT mice as part of FMT assays. Our findings revealed the substantial upregulation of sets of genes in mice that received Tg feces compared to those receiving WT feces, among which the expression of *Saa1* that encodes SAA was the most affected one (Fig. 2a; Supplementary Fig. S3a). Meanwhile, *Saa2* and *Saa3*, encoding two other SAA isoforms, were also upregulated (Fig. 2b; Supplementary Fig. S3b, c).

SAA is an apolipoprotein that closely interacts with lipoproteins including high-density lipoprotein (HDL), low-density lipoprotein (LDL), and very low-density lipoprotein (VLDL)^25–27^. In line with the SAA level change, we also noted significant upregulation of genes enriched in functional pathways such as HDL and phospholipase activity, and the downregulation of pathways related to the negative regulation of immune functions^27^ (Supplementary Fig. S3d–g). SAA is originally known to be excreted by hepatic cells^28–30^. To understand whether SAA originated in the ileum in this context, ileal tissues of mice receiving feces of 41-week-aged Tg mice were subjected to immunohistochemistry (IHC) staining. We discovered strong SAA protein staining at the tip of the villus in ileum tissue of the mice that received feces from Tg vs from WT (Fig. 2c). This result indicated that, in addition to liver and other extrahepatic cells^28–30^, gut epithelial cells also contributed to SAA production, likely in context-dependent manners.

SAA is mainly known to act as one of the acute phase reaction proteins that are involved in inflammatory responses and to activate the innate immune cells like monocytes^31^. As such, we treated human THP-1 monocytes with human SAA (hSAA). Results showed that hSAA could dose-dependently stimulate the activation of NF-κB signaling in THP-1 cells, indicating the likely of THP-1 activation (Supplementary Fig. S4a). Analysis of cytokine array data further revealed a significant increase in a wide range of cytokines upon exposure to hSAA, including IL-6 and TNF-α, key indicators of THP-1 activation (Fig. 2d; Supplementary Fig. S4b). Of note, we also observed enhanced production of IL-12 and IFN-γ, two cytokines that promote Th1 cell differentiation (Supplementary Fig. S4c). All these prompted us to hypothesize that SAA-stimulated monocytes might account for Th1 cell differentiation. To test this hypothesis, we conducted a co-stimulation experiment in which THP-1 cells were exposed to SAA and subsequently co-cultured with naive CD4^+^ T cells, and naive CD4^+^ T cells treated yet without THP-1 co-culture was used as a control. We found that SAA per se, without THP-1 co-culture, could not activate naïve CD4^+^ T cells to differentiate into Th1 cells (Supplementary Fig. S4d). Only under co-culture condition, SAA-stimulated THP1 cells could significantly increase Th1 frequency compared to the vehicle-stimulated cells (Fig. 2e). We also examined the effect of SAA on dendritic cells (DC). Similar results were observed using mouse bone marrow-derived dendritic cells (BMDCs). Mouse SAA (mSAA) increased the production of multiple pro-inflammatory cytokines, including DC and Th1 inducers such as RANTES, TNF-α, G-CSF, IL-6, and IL-12p40, along with decreased Th2 inducer IL-4 (Fig. 2f).

We then examined the blood SAA level and Th1 cell frequency in vivo. For this, we selected Tg mice aged at 41 weeks and used age-matched WT mice as control. We found that both SAA level and Th1 cell frequency were higher in the plasma of Tg mice as compared with those of WT mice (Fig. 2g, h), suggesting the involvement of SAA in Th1-skewed inflammation in Tg mice. Our previous study has shown that the increased peripheral Th1 differentiation and proliferation in Tg mice were due to the gut microbiome change^13^. We then asked whether the observed changes were also caused by the imbalanced gut microbiota. For this, we transplanted the mice with Tg feces. We found an increased trend in blood SAA levels and a significant increase in monocytes and Th1 cell frequency, as compared to those transplanted with WT feces (Fig. 2i–k), indicating that SAA and immune cell changes could be induced by the imbalanced gut microbiota.

### *L.m*. strain derived from Tg mice is characterized with high expression of Rib compared with that from WT Mice

**Fig. 3:**
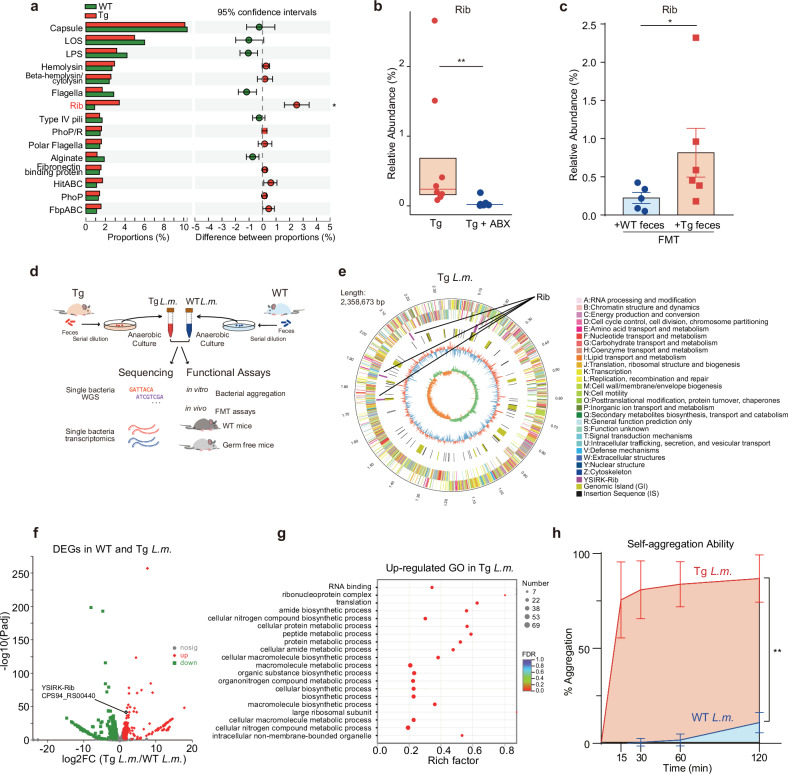
*L.m*. derived from Tg mice feces vs WT mice feces harbors higher expressed *Rib* gene and is associated with deregulated metabolites and enhanced aggregation phenotype. **a** Changes in the relative abundances of the top 15 VFs of WT and Tg mice feces at w41 from the VFDB (*n* = 4). Data are represented as mean ± SD. *P < 0.05, by Student’s *t*-test. **b** Changes in the relative abundances of the Rib of the gut microbiota of 7-month-old Tg and ABX-treated Tg mice. Box plots represent the median (the horizontal line within boxes) and the 75^th^ and 25^th^ percentiles (the top and bottom of each box, respectively). The upper and lower whiskers represent 1.5× IQR from the top and bottom of the box, respectively. **P < 0.01 by Wilcoxon rank-sum test, *n* = 8. Tg: *n* = 8, 4 M + 4 F; Tg + ABX: *n* = 8, 4 M + 4 F. M, male mice; F, female mice. **c** Changes in the relative abundances of the Rib of the gut microbiota of 7-month-old Tg recipient mice that received w41 WT (+WT feces) and Tg feces (+ Tg feces) in FMT assays. Data are represented as mean ± SEM. *P < 0.05, by Wilcoxon rank-sum test, *n* = 5–6. **d** Schematic design of experiments to understand the differences between *L.m*. species isolated from 41-week-age WT and Tg feces. After collecting the feces from both mice, serial dilution was applied and further cultured on the MRS selection plate under an anaerobic chamber for the enrichment of *L.m*. species. PCR-guided sequencing was used to validate the bacteria’s identity. After that, we cultured the individual species to the logarithm phases to obtain rapidly propagating species and then performed single-base whole genome sequencing and transcriptomics. Functional assays were further designed to compare the function of these species, including the in vitro aggregation test, and the in vivo FMT assays using either WT mice or GF mice. **e** Genomic map of Tg-*L.m*. sequenced by bacteria complete map sequencing and plotted using Circos software. Outer ring to inner ring signifies information of genome size (number in megabase pair, Mbp), genomic information of coding sequence (CDS) area on positive chain and negative chain (color labels showing different clusters of orthologous group (COG) function of CDS), location of Rib (purple line), location of genomic island (GI, in yellow line), location of insertion sequence (IS, in black line), GC% contents (outer red or inner blue bar indicating that the GC% of this area is higher or lower than the average GC content of the whole genome. The higher the bar, the larger the differences), and GC-Skew value (or (G − C)/(G + C) value used to define leading and lagging strands). **f** Volcano plots of DEGs in WT and Tg-*L.m*. sequenced by single-bacteria transcriptomics. The *x* and *y* axes represent log_2_FC of genes in Tg-*L.m*. vs WT-*L.m*. and the negative log_10_ of *P*_adj_ of these genes. Red, up-regulated in Tg*-L.m*.; green, down-regulated in WT*-L.m*.; gray, not significant (nosig); Rib, the Rib repeats. **g** Gene ontology (GO) enrichment analysis of up-regulated genes in Tg-*L.m*. vs WT-*L.m*. FDR, false-discovery rate. **h** In vitro bacteria aggregation ability of *L.m*. bacteria isolated from WT and Tg mice feces at 15–120 min. **P < 0.01, by Unpaired *t*-test, *n* = 3.

We next aimed to identify specific microbial species that could dominantly contribute to the aforementioned molecular changes. Our particular focus centered on *L.m*., the significantly increased species in Tg mice. It is widely recognized that an increase in microbiota abundance can enhance host–microbe interaction, which is facilitated by bacterial adhesins that enable the adherence of bacteria to host epithelial cells, leading to subsequent downstream effects^32–35^. As adhesins are one type of virulence factors (VFs), we mapped our metagenomics data to the virulence factor database (VFDB) to identify candidate VFs in the 41-week Tg mouse fecal samples. Notably, within the group of highly abundant VFs, a putative surface adhesin that included YSIRK signal domain/LPXTG anchor domain surface protein and Rib/alpha/Esp surface antigen was the only one to show a significant upregulation in Tg vs WT, and was named Rib (resistance to proteases, immunity, group B^36^) (Fig. 3a; Supplementary Table S1). We further found that the proportions of Rib increased significantly along disease progression in Tg mice, while WT mice did not show this correlation (Supplementary Fig. S5a). To confirm whether there existed some connection between the adhesin and gut microbiota, we manipulated gut microbiota utilizing ABX and FMT approaches, respectively. We found that eliminating the gut microbiota in Tg mice with ABX significantly decreased the fecal levels of the Rib (Fig. 3b). Conversely, mice that received Tg fecal samples exhibited significantly higher levels of Rib compared to those receiving WT fecal samples (Fig. 3c).

Rib is reported to be the adhesion molecule of the *Lactobacillus* species^36^. We hence investigated whether variations in Rib are associated with specific *L.m*. strains isolated from WT and Tg feces. To this end, we employed an integrated approach comprising multi-omics sequencing and functional assays on these isolated strains (Fig. 3d). PCR-guided techniques isolated the significantly increased *L.m*. strain from the 41-week Tg and WT mouse feces, and both were then subjected to the multi-omics sequencing. We found distinct genomic variations between the Tg-derived *L.m*. (Tg-*L.m*.) and the WT-derived *L.m*. (WT-*L.m*.). The genome size of Tg-*L.m*. was 2,358,673 bp, whereas that of WT-*L.m*. was 2,244,140 bp (Fig. 3e; Supplementary Fig. S5b). Importantly, the two *L.m*. strains exhibited differences in various genomic features, including the number of genomic islands, the overall length of prophages, and number of insertion sequences (Supplementary Table S2). Moreover, the two strains also exhibited variations in functional attributes, including the number of genes annotated as carbohydrate-active enzymes (CAZyme) and secondary metabolite biosynthetic gene clusters (smBGCs), etc. (Supplementary Table S2). Notably, there were different numbers of predicted VFs between Tg-*L.m*. and WT-*L.m*. (Supplementary Fig. S5c). Among the VFs, *Rib* gene was indeed encoded in the chromosomes of both *L.m*. strains. Further insights demonstrated that the *Rib* gene revealed features typical of Gram-positive surface proteins that were covalently anchored to the cell wall, including the YSIRK signal sequence, an LPxTG anchor motif, and a Rib repeat region responsible for adherence^37^. It has been reported that the component of the gene encoding *Lactobacillus* adhesin actually varies greatly among different species with very different molecular weights, numbers and types of the repeat domain, and different combinations of YSIRK with the repeat domain as well as the anchor motif^38^. In line with this, we observed that the Rib genes in Tg-*L.m*. and WT-*L.m* differed from each other in either gene length, chromosomal location, or strand orientation. Moreover, different numbers of Rib repeats were also observed in two strains (Supplementary Table S3).

Next, we moved to assess whether Rib was actively transcribed. For this, we conducted single bacterial RNA-seq. We observed significant differences in overall gene expression between Tg-*L.m*. and WT-*L.m*. As anticipated, Rib (CPS94_RS00440) was significantly upregulated in Tg-*L.m*. compared to WT-*L.m*. (Fig. 3f; Supplementary Table S4). Along with this, the functional enrichment analysis of the up-regulated genes in Tg-*L.m*. further revealed multiple metabolic-related pathways that were highly involved in Tg-*L.m*., including protein translation, amide biosynthetic process, and macromolecule metabolic and biosynthetic process, as compared with that of WT-*L.m*. (Fig. 3g). Furthermore, we noted a significantly stronger self-aggregation ability in Tg-*L.m*. strain than that of the WT-*L.m*. (Fig. 3h), suggesting a stronger adherence capacity in Tg-*L.m*. strain. These findings substantiated that though WT-*L.m*. and Tg-*L.m*. belonged to the same *L.m*. species, the two were functionally quite different. The differential genomic characteristics of Rib in each strain, combined with the observed differences in gene expression and aggregation phenotypes, underscored the variance in the virulence potential between the two strains. Whether the disparity between the two strains is driven by genetic factors requires further investigation.

### Tg-derived Rib^high^-*L.m*. induces significant metabolic changes, activation of SAA, and Th1-skewed inflammation

**Fig. 4:**
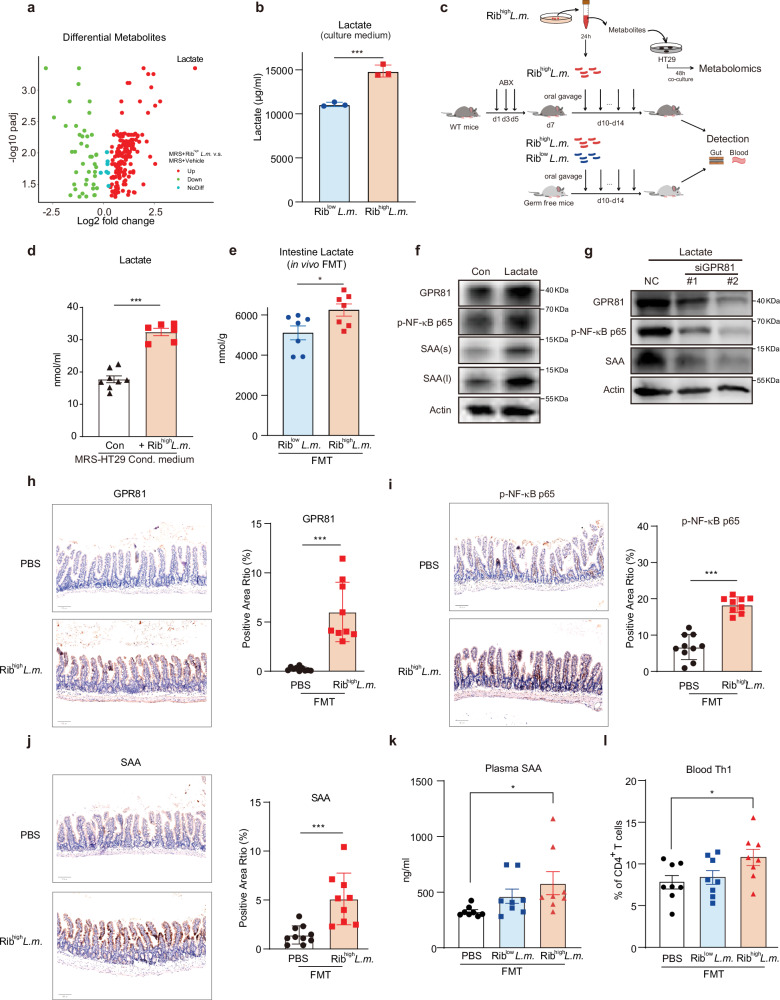
Lactate activates SAA via the GPR81-NF-κB pathway. **a** FC and *P*_adj_ of the levels of differential metabolites of MRS-broth cultured with Tg-*L.m*. (Rib^high^-*L.m*.) compared with MRS-broth alone without bacteria detected by untargeted metabolomics. Color legends indicate up-regulate (red) or down-regulate (green) metabolites in MRS-broth cultured with Tg *L.m*. (MRS + Rib^high^-*L.m*.); noDiff, no difference between groups. **b** Lactate level in the 24-h anaerobic MRS culture medium of Rib^low^-*L.m* and Rib^high^-*L.m*. ***P < 0.001 by Student’s *t*-test. **c** Schematic design of in vitro and in vivo assays on host–bacteria interaction using Rib^high^-*L.m* and Rib^low^-*L.m*. **d** Lactate changes in the 48 h *L.m*. MRS-HT29 condition medium (+Rib^high^-*L.m*.) vs the MRS-HT29 condition medium without *L.m*. culture (Con). ***P < 0.001 by Student’s *t*-test. **e** The in vivo intestine lactate levels of the recipient GF mice that received FMT of single Rib^low^-*L.m*. and Rib^high^-*L.m*. species. * *P* < 0.05, by Student’s *t*-test. **f** Effect of 10 mM lactate on the protein level of GPR81, phosphorylated NF-κB p65 (p-NF-κB p65), and SAA in HT29 cells. Cells were treated with lactate for 2 h followed by cell lysis and western blotted with the indicated antibodies. s, short exposure; l, long exposure. **g** Effect of transient knockdown of GPR81 (small interference RNA, or si*GPR81*) on the protein level of GPR81, p-NF-κB p65, and SAA in HT29 cells that stimulated by 2 h treatment of 10 mM lactate. All siRNAs were added to HT29 cells with RNAimax 6 h before stimulating by lactate. After 2 h stimulation, HT29 cells were subjected to western blotting with the indicated antibodies. **h**–**j** Ileum IHC staining results of GPR81 (**h**), p-NF-κB p65 (**i**), and SAA (**j**) of the recipient WT mice transplanted with two-week oral gavage of 1 × 10^9^ CFU/mL single-bacteria suspension of Rib^high^-*L.m*. Representative images were shown on the left, with statistical results shown on the right. ***P < 0.001, by Student’s *t*-test. *n* = 9–10 per group. **k** Plasma SAA levels from the recipient germ-free (GF) mice transplanted with two-week oral gavage of 1 × 10^9^ CFU/mL single-bacteria suspension of Rib^low^-and Rib^high^-*L.m*., detected using ELISA. PBS was given the same volume as bacteria suspension as control. *P < 0.05, by Mann–Whitley test, *n* = 8 per group. **l** Blood Th1 cell frequency of the recipient GF mice transplanted with two-week oral gavage of 1 × 10^9^ CFU/mL single-bacteria suspension of Rib^low^-and Rib^high^-*L.m*. PBS was given the same volume as bacteria suspension as control. *P < 0.05, by Mann–Whitley test, *n* = 8 per group. All recipient mice in FMT assays are male mice. Data are represented as mean ± SEM.

It is widely acknowledged that an increase in the abundance of gut bacteria can allow the bacteria to adhere to host epithelial cells via adhesins and preferentially lead to subsequent metabolic reprogramming. We then asked whether the increased abundance of Tg-*L.m*. strain, featured by higher Rib expression (termed Rib^high^-*L.m*. thereafter) and stronger ability of self-aggregation, was able to trigger the metabolic disorders per se. To this end, we cultured Rib^high^-*L.m*. in an anerobic setting. Using untargeted metabolomics, we found that Rib^high^-*L.m*. by itself released a significant number of metabolites with lactate ranking the top, as compared with culture medium without Rib^high^-*L.m*.(Fig. 4a; Supplementary Table S5), indicating that Rib^high^-*L.m*. was critical for lactate production. Indeed, Rib^high^-*L.m*. exhibited a significantly higher lactate level either within the culture medium or within the bacteria as compared to that of Rib^low^-*L.m*. (Fig. 4b; Supplementary Fig. S5d). All these highly suggested that Rib^high^-*L.m*. was intrinsically empowered with increased ability to generate lactate than Rib^low^-*L.m.*

Given that the bacteria–host interaction plays an indispensable role in modulating metabolic outputs^17,39^, we then applied both in vitro co-culture assays and in vivo FMT assays that allowed the stringent bacteria–host interaction (Fig. 4c). For in vitro host-bacteria co-culture, we selected HT29 cells, a model human intestinal epithelial cell line^40^, to be cultivated with the medium of Rib^high^-*L.m*. Along with significantly increased phenylalanine and isoleucine, which aligned with previous findings^13^ (Supplementary Fig. S5e, f), we observed that co-culture of Rib^high^-*L.m*. with HT29 cells resulted in significant increase of lactate (Fig. 4d). Meanwhile, transplantation of Rib^high^-*L.m*. into germ-free (GF) mice could also lead to significantly higher levels of intestinal lactate as compared with that in Rib^low^-*L.m*. colonized GF mice (Fig. 4e). All these collectively depicted that upon host–bacteria interaction, Rib-mediated epithelium adherence triggers metabolic reprogramming and facilitates lactate production.

Lactate is known to activate GPR81, and GPR81 is known to activate NF-κB signaling^41–43^, while NF-κB can transcriptionally activate SAA^44,45^. We then asked whether lactate participates in activating SAA production via GPR81-NF-κB axis. To test this assumption, we treated HT29 cells with lactate^42^. We found that exposure of HT29 cells to lactate led to the activation of GPR81, p-NF-κB p65, and SAA production simultaneously (Fig. 4f). Similar effects were obtained using 3-chloro-5-hydroxybenzoic acid (3-Cl-5-OHBA), a GPR81 agonist^46^ (Supplementary Fig. S5g). To examine whether GPR81 was involved in mediating the effect of lactate in this setting, we used siRNA to knockdown GPR81. As expected, GPR81 knockdown significantly impeded the SAA production induced by either lactate or 3,5-dihydroxybenzoic acid (3-5-DHBA), another GPR81 agonist (Fig. 4g; Supplementary Fig. S5h).

We next asked whether Rib^high^-*L.m*. could activate the aforementioned signaling in gut epithelium and increase SAA and Th1 levels in the blood. To this end, we conducted FMT to transplant Rib^high^-*L.m*. strains into recipient mice. Consistent with the in vitro findings, Rib^high^-*L.m*. colonization could significantly increase the levels of GPR81, p-NF-κB p65 and SAA in the ileum epithelial cells (Fig. 4h–j). Moreover, Rib^high^-*L.m*. could also induce a significant increase of plasma SAA and Th1 cell in the recipient GF mice (Fig. 4k–l), strengthening a notion that Tg-derived Rib^high^-*L.m*. enabled the stimulation of SAA production and Th1 cell activation in vivo.

### Elevated genomic abundances of Rib in AD patients correlate with the increased lactate and SAA levels

**Fig. 5:**
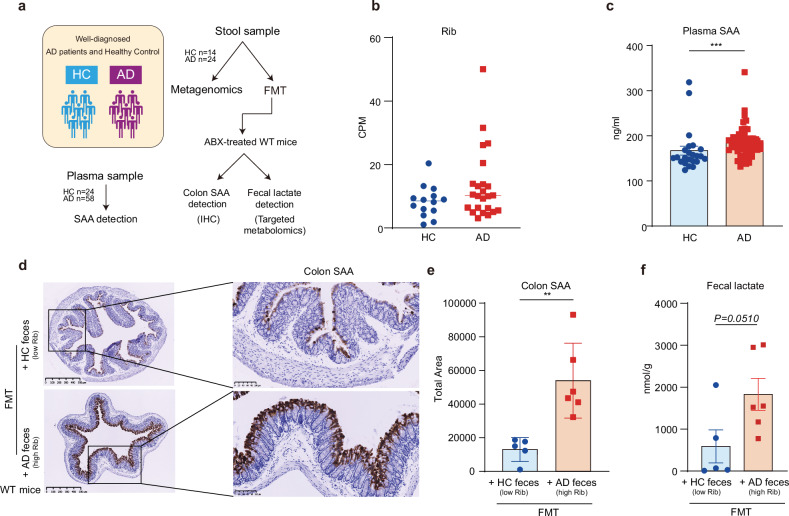
The correlation among Rib, lactate, and SAA in a small cohort of AD patient samples. **a** Schematic outline of patient selection and validation tests workflow. Stool and plasma samples from health control (HC) and AD patients were collected after clinical diagnosis including cognition test, neuroimaging using magnetic resonance imaging (MRI), and blood test on a series of AD markers including Aβ 42/40, total-Tau, NFL, and GFAP, etc. Plasma samples were tested for SAA using ELISA, while stool samples were collected for metagenomics sequencing and FMT assays on ABX-treated WT mice. Colon SAA of the recipient mice was detected via antibodies targeting SAA using IHC and was statistically analyzed by an experienced pathologist on positive-staining areas. The fecal lactate of the recipient mice was detected using targeted metabolomics. **b** Relative abundance of Rib in the gut feces samples from HC and AD patients. CPM, Counts per million mapped reads. HC group: *n* = 14, AD group: *n* = 24. **c** Plasma SAA level of HC and AD patients detected by ELISA. HC group: *n* = 24, AD group: n = 58. ***P < 0.001, by Mann–Whitney test. **d**, **e** Representative IHC staining images (**d**) and positive area statistics (**e**) of colon SAA from the recipient ABX-treated WT mice transplanted with feces samples from AD patients with high Rib and HC subjects with low Rib abundances in FMT assays. *n* = 5–6, **P < 0.01, by Student’s *t*-test. **f** Levels of fecal lactate level from the recipient ABX-treated WT mice transplanted with feces samples from AD patients with high Rib and HC subjects with low Rib abundances in FMT assays. *n* = 5–6, *P* = 0.0510, by Student’s *t*-test. The high and low Rib abundances were chosen by ranking, i.e., we chose the top 6 samples with the highest Rib abundances in AD feces and the bottom 5 samples with the lowest Rib abundances in HC feces. All recipient mice in FMT assays are male mice. Data are represented as mean ± SEM.

Subsequently, we sought to determine whether our findings observed in Tg mice could be confirmed in AD patients. For this, 58 clinically diagnosed AD patients and 24 healthy controls (HC) were included in the study. Among them, 24 AD patients and 14 HC individuals provided fecal samples (Fig. 5a), from which we identified 9 bacterial species containing the Rib domain that most of them were more abundant in AD patients compared to HC, though these bacteria are relatively low in overall abundance (Supplementary Fig. S5i). Given that *L.m*. is exclusive to mice and together with the fact that Rib is a bacterial adhesin commonly present across multiple species^47^, we then focused on Rib domain instead of specific bacteria species by evaluating the abundance of Rib gene sequences in the fecal samples using metagenomic data. We found that AD patients exhibited higher Rib abundance compared to that of HC (Fig. 5b), along with significantly higher levels of SAA in their blood as compared to HC (Fig. 5c).

Further, we asked whether fecal samples from AD patients with higher Rib could induce gut epithelial SAA production. To this end, fecal samples from AD patients exhibiting higher Rib abundances were transplanted into WT recipient mice, and those from HC subjects with lower levels of Rib were used as control. We observed the increased colon SAA staining in mice receiving feces from AD patients characterized by higher Rib abundances (Fig. 5d, e). In these mice, we detected a significant elevation in the level of fecal lactate as well (Fig. 5f). These observations reinforced the causal link between Rib^high^ bacteria and enhanced SAA production in gut and increased level of fecal lactate in AD patients.

### GV-971 directly binds to GIANLDKL domain of Rib to reduce bacterial adhesion to gut epithelia

We previously demonstrated that GV-971 could rectify gut microbiota imbalances to harness neuroinflammation, yet how GV-971 remodels the gut microbiome is unclear. GV-971 consists of acidic 6-carbon sugar rings connected to poly-hydroxyl tail(s) structurally, similar to that of sialic acid. As Rib belongs to a large family of the *L.m*. adhesins that recognize terminal sialic acid residues in gut mucus (mucin)^36,48^, we thus hypothesized that GV-971 may compete with sialylated mucin-adhesin interaction and thus prevent the bacteria adhesion to the host^49–51^. To test this hypothesis, we took an integrative approach combining sequence analysis, 3D structure modeling, virtual docking, in vitro protein binding assays, and in vivo tests to probe the binding potential of GV-971 to Rib.

**Fig. 6:**
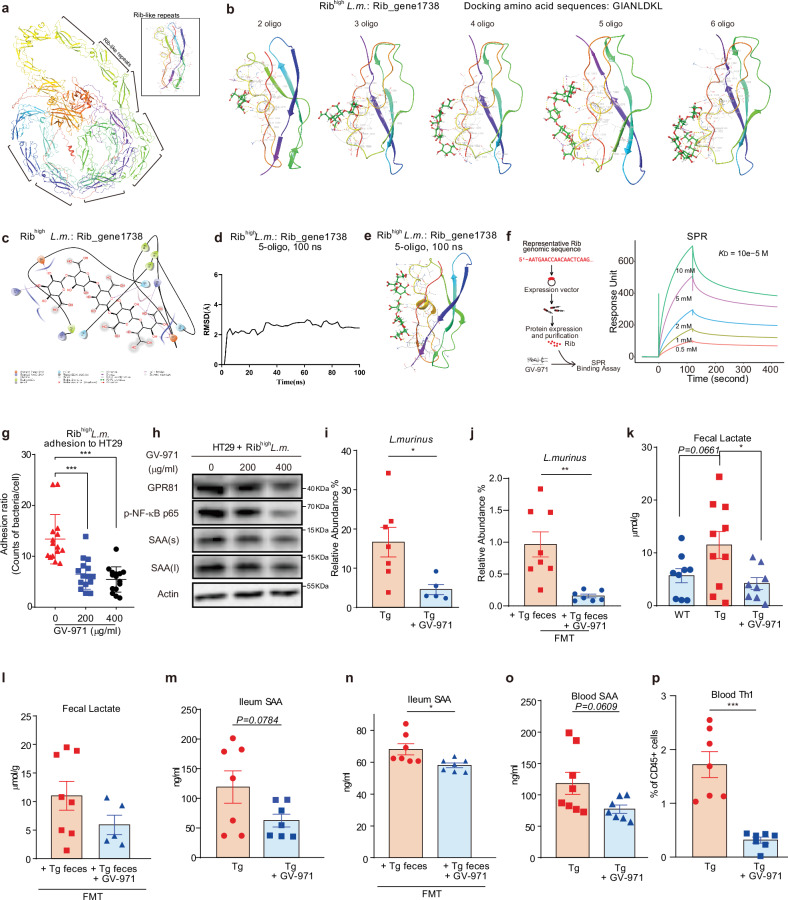
Binding effect of GV-971 on Rib adhesion domain and its inhibitory effect on *L.m*. adhesion, lactate-SAA pathway, and Th1 activation. **a** Protein structure of the Rib domain from *L.m*., which we named Rib_gene1738. The black bracket demonstrates the protein structure of one representative Rib repeated domain, of which the repeated GIANLDKL residues were present (also see Supplementary Fig. S6a). **b** Detailed interaction of 2 to 6 sugar units of GV-971 (solid sticks) with repeated GIANLDKL residues of the Rib_gene1738 (color ribbon) protein from Rib^high^-*L.m*. The binding sites are shown. Hydrogen bonds and salt bridges are depicted as yellow and purple dashed lines, respectively. Different oligomers of GV-971 are shown as sticks. The repeated GIANLDKL residues are displayed in green, orange, and blue ribbons. **c** Type of connection between GV-971 and GIANLDKL residues of the Rib_gene1738 protein (color ribbon) from Rib^high^-*L.m*. **d**, **e** Molecular dynamics simulations of 5 sugar units of GV-971 interacting with repeated GIANLDKL residues of the Rib_gene1738 protein (color ribbon) from Rib^high^-*L.m*. using AMBER at total 100 nanoseconds (ns) simulation. RMSD values through 100 ns (**d**) and interacting forces including hydrogen bonds and salt bridges are depicted as yellow and purple dashed lines, respectively (**e**). **f** SPR sensor grams of the Rib_gene2117-GV-971 interaction. Concentrations of GV-971 containing 2–10 sugar units (from bottom to top) are 0.5, 1, 2, 5, and 10 mM, respectively. *K*_D_ value is 10^−5 ^M. **g** The effect of GV-971 of two different doses (200 μg/mL and 400 μg/mL, final concentration) on the adhesion of Rib^high^*-L.m*. to HT29 cells at 2-h incubation time. The numbers of bacteria per HT29 cells were calculated in 4 to 5 separate areas for each well and replicated 3 times. Data are represented as mean ± SD. ***P < 0.001 by Student’s *t*-test, *n* = 15. **h** The effect of GV-971 of two different doses (200 μg/mL and 400 μg/mL, final concentration) on GPR81-NF-κB signaling and SAA levels of HT29 cells in adhesion assays in **g**. s, short exposure; l, long exposure. **i** The effect of 100 mg/kg GV-971 on the relative abundance of the 41-week-age Tg mice gut microbiota species *L.m*. Data are represented as mean ± SEM. *P < 0.05 by Student’s *t*-test, *n* = 5–7. Tg: *n* = 7, 3 M + 4 F; Tg+GV-971: *n* = 5, 3 M + 2 F. M, male mice; F, female mice. **j** The relative abundance of fecal *L.m*. in the WT recipient mouse that received 40-week-age Tg mouse feces treated or not treated by 100 mg/kg GV-971. Data are represented as mean ± SEM. **P < 0.01 by Student’s *t*-test, *n* = 7–8. **k** Effect of 100 mg/kg GV-971 on fecal lactate levels of the 41-week-age Tg mice feces detected by targeted metabolomics. Data are represented as mean ± SD. *P < 0.05 by Student’s *t*-test, *n* = 8–10. **l** The relative abundance of fecal lactate in the WT recipient mouse that received 40-week-age Tg mouse feces treated or not treated by 200 mg/kg GV-971. The levels of lactate were detected by targeted metabolomics. Data are represented as mean ± SEM, *n* = 5–8. **m** Effect of one-month treatment of 100 mg/kg GV-971 on Ileum SAA levels of 9-month-old Tg mice. Data are represented as mean ± SEM, *n* = 7. Tg: *n* = 7, 7 M; Tg+GV-971: *n* = 7, 5 M + 2 F. M, male mice; F, female mice. **n** The relative abundance of ileum SAA in the WT recipient mouse that received 40-week-age Tg mouse feces treated or not treated by 200 mg/kg GV-971. Data are represented as mean ± SEM. *P < 0.05 by Student’s *t* test, *n* = 7. **o** Effect of 100 mg/kg GV-971 on blood SAA levels of the 41-week-age Tg mice. The levels of SAA were determined by ELISA. Data are represented as mean ± SEM, *n* = 7–8. Tg: *n* = 8, 4 M + 4 F; Tg + GV-971: *n* = 7, 4 M + 3 F. M, male mice; F, female mice. **p** Effect of 100 mg/kg GV-971 on blood Th1 cells of the 41-week-age Tg mice. Data are represented as mean ± SEM. ***P < 0.001 by Student’s *t*-test, *n* = 7. Tg: *n* = 7, 7 M; Tg+GV-971: *n* = 7, 5 M + 2 F. M, male mice; F, female mice. All recipient mice in FMT assays are male mice.

As Rib structure is not well documented in *L.m*., we used the AlphaFold2 structure prediction algorithm to simulate the structures of all Rib paralogs proteins identified in bacteria de novo sequencing of Rib^high^-*L.m*. Here, we chose the Rib_gene1738 as a representative and found many repeat domains surrounding the protein, with a three-dimensional structure resembling the adhesive Rib domain, or ‘Rib Long’, from *Lactobacillus acidophilus* (PDB: 6S5Y, 6S5w) (Fig. 6a). All these implicated that these repeat domains may account for binding properties. Among these repeats, we further identified the amino acid fragment GIANLDKL as an optimal docking motif based on its resemblance to the modeling fragment from other bacteria (Supplementary Fig. S6a)^47^. Then, we used GIANLDKL repeats and established 1376 different GV-971 ligands conformations. Among these conformations, seven GV-971 conformations performed the best scores by the glide-docking algorithm (Supplementary Fig. S6b). Furthermore, molecular docking displayed a stable binding between GV-971 and GIANLDKL of *L.m*. bacterial Rib_gene1738 protein (Fig. 6b, *n* = 2–6 sugar unit of GV-971). Further analysis revealed that the molecular forces of GV-971 binding to GIANLDKL repeats of *L.m*. bacterial Rib_gene1738 were mainly hydrogen bonds (Fig. 6c).

To simulate the binding changes in the aqueous state, we used the molecular dynamics software AMBER^52^ which adopts the theoretical model using one ligand molecule plus one receptor molecule. We found that in the 100 complex trajectory calculation, the tested 5 sugar units of GV-971 could bind to the protein with good stability. The fluctuation of root-mean-square deviation (RMSD) was within the 1 Å range (Fig. 6d, e), indicating the stable binding settings in this scenario. We also found that other Rib domains, including Rib^high^-*L.m*. Rib_gene0115, Rib_gene1850, Rib_gene2116, and Rib_gene2117, displayed similarly stable static and dynamic binding profile to GV-971, yet the exact binding sites varied according to different structures of Rib paralogs (Supplementary Fig. S6c–f).

The computational simulation results prompted us to hypothesize that GV-971 could directly bind to these proteins. To test this possibility, we used surface plasmon resonance (SPR) to measure the binding affinity of GV-971 for Rib. To achieve this, we selected Rib_gene2117 as a representative protein, which contains the GIANLDKL repeats and can be easily expressed in the *Escherichia coli* expression system (Fig. 6f, left panel). We purified the full-length protein of Rib_gene2117 and immobilized it on a chip. Then, GV-971 sugar units at various concentrations were injected over a full-length Rib immobilized chip. Interestingly, GV-971 sugar units were able to bind to the immobilized protein in a concentration-dependent manner and exhibited clear exponential curves in both the association and dissociation phases, with a *K*_D_ value of 10^−5 ^M (Fig. 6f, right panel).

### GV-971 reduces ileum epithelial SAA production and suppresses Th1 activation in Tg mice

We next investigated the impact of GV-971 on the adhesion of Rib^high^-*L.m*. to gut epithelium. For this, Rib^high^-*L.m*. was incubated with HT29 cells in the presence or absence of GV-971. We found that GV-971 at given concentrations significantly reduced the number of Rib^high^-*L.m*. bacteria adhering to HT29 cells (Fig. 6g). Accordingly, GV-971 treatment decreased GPR81 expression, inhibited p-NF-κB p65 signaling, and reduced SAA production in HT29 cells in a dose-dependent manner (Fig. 6h). In addition, we noted that GV-971 could significantly reduce the levels of phenylalanine and isoleucine in HT29 cells in the co-culture assay, consistent with our previous findings (Supplementary Fig. S7a, b).

To test whether the effects of GV-971 observed in vitro could be recapitulated in vivo, we moved to examine the impact of GV-971 on *L.m*. abundance, lactate levels, SAA production, and Th1 cell frequency in Tg mice sequentially. First, we focused on the effect of GV-971 on *L.m*. abundance. Metagenomics analysis revealed that GV-971 treatment at a given dosage significantly decreased the relative abundance of *L.m*. in Tg mice (Fig. 6i). In mice that received Tg feces, GV-971 treatment could also significantly decrease *L.m*. abundance (Fig. 6j). Then we investigated the effect of GV-971 on lactate level. We found that GV-971 significantly corrected excessive fecal lactate levels in Tg mice to levels comparable to WT (Fig. 6k) and resulted in a trend towards reducing lactate in mice that received Tg feces (Fig. 6l). Moreover, GV-971 treatment inhibited the production of ileum SAA in Tg mice (Fig. 6m, n), with a more prominent effect observed in models from FMT experiment (Fig. 6n). Besides ileum SAA, GV-971 could also significantly decrease blood SAA levels by about 30%, compared to untreated group (Fig. 6o). Further, we found that GV-971 significantly reduced Th1 cell frequency in the blood (Fig. 6p), which was consistent with our previous finding^13^. Taken together, our data made an advance in revealing the mechanism of action of GV-971. It interferes with Rib^high^-*L.m*. adhesion to ileum epithelial cells by directly interacting with the functional domains of Rib adhesins, which attenuates SAA-driven Th1 immune responses in vivo.

## Discussion

In our previous study, we reported that dysbiosis of gut bacteria leads to a reshaping of metabolic reprogramming in transgenic models^13^. We and others also reported that GV-971, a marine-derived mixture of oligosaccharides restored the balance of gut microbiota and positively affected metabolic processes^13,14^. Despite these intriguing findings, there remains a lacuna in our understanding of how dysbiosis of gut flora, and the specific species, are responsible for reconstituting metabolic reprogramming and how GV-971 interferes with the gut microbiome. In this study, we made significant advances by showing that the gut microbiome of 5XFAD Tg mice was enriched in bacteria expressing Rib VFs, which facilitated the attachment of gut bacteria to gut epithelial cells, resulting in a reconstitution of metabolic reprogramming. These findings were replicated in AD patients, suggesting the possibility that Rib^high^ bacteria could be involved in AD progression.

Moreover, we provided the first evidence showing that GV-971 binds specifically to the GIANLDKL repeats of Rib. We show that GV-971 binds to the GIANLDKL repeats of Rib adhesin mainly via hydrogen bonds. GV-971, with its acidic 6-carbon sugar rings connected to a poly-hydroxyl tail, structurally resembles sialic acid. It is acknowledged that highly sialylated gut epithelial mucin is one of the key players involved in the adhesion by gut bacteria via their surface virulent proteins to gut epithelial cells^36,48^. The presence of exogenous sialic acid-like sugar chains in GV-971 can compete with the natural sialic acid, thus interfering with the adhesion process. This particular mechanism of action allows the susceptibility of Rib^high^ bacteria to GV-971 and explains how GV-971 interacts with the gut microbiome in AD mice.

The communication between bacteria and the host can result in changes to metabolic pathways and alterations in the host’s metabolomics, which can contribute to the development of various diseases including AD^53^. We previously discovered phenylalanine and isoleucine as two essential amino acids accounting for peripheral pro-inflammation associated with AD. However, it remains unclear whether other metabolites due to metabolic reprogramming settings may be involved in this process, since various metabolites can function as signaling molecules involved in proinflammation milieu. In the current study, we identified stage-specific alterations in metabolites in 41-week-old Tg mice, with excess lactate being particularly notable in Tg vs WT feces. Lactate plays a physiological role in providing energy to brain astrocytes for neuron survival^54^ and has been known to regulate vasculature development and progenitor behavior in the mouse neocortex through CXCL1 signaling^55^. However, excessive lactate release can have harmful effects^56^, which largely depends on lactate homeostasis in different contexts^57^. Generally, in this study, we showed that excess lactate functions as the signal molecule to stimulate the epithelial production of SAA via the GPR81-NFκB axis, consequently contributing to the activation of peripheral Th1 cells. All these findings show that different metabolites with different mechanisms of action may share common outcomes linking immune activation, such as Th1-skewed proinflammation, in AD progression.

Importantly, we identified previously unrecognized, non-canonical SAA production in the gut of AD. While SAA is traditionally known as an acute-phase response protein produced in the liver in response to bacterial stimuli, recent studies have shown that gut epithelial cells can also produce SAA in response to segmented filamentous bacteria (SFB)-induced STAT3 activation or flagellins from SFB^58,59^. Our findings provide evidence that gut epithelial cells are indeed one of the sources of SAA production and that SAA production can be triggered by lactate-sensing signaling. This adds to our understanding of the critical role of SAA in chronic or recurrent inflammatory settings and provides a potential link to the systemic inflammation in AD. As SAA can interact with innate immune cells^59,60^, our discovery that SAA can activate innate immunity and subsequent Th1-skewed immune response suggests a possible role for SAA in immune regulation during AD progression. Besides, the deregulated SAA during chronic inflammation could be attributed to various VFs, inflammatory factors, and metabolite sensing cascades, which needs further investigation.

Moreover, we suggested that SAA is associated with HDL related pathway (Supplementary Fig. S3e). Together with other reports mentioned above, the SAA-HDL relationship has linked the development of dementia to HDL. Strikingly, a very recent study has illustrated the association of plasma HDL-cholesterol level with risk of incident dementia^61^, highly suggesting a mechanistic link among gut microbes, metabolic reprogramming, SAA, lipoprotein metabolism, and Th1 skewed peripheral inflammation in AD development. It is also worthwhile to evaluate whether SAA in the gut can serve as a potential therapeutic target for intervention in AD. Overall, the specific involvement of these factors may vary depending on the disease stage and tissue type, and it is important to note that although sex differences are often observed in the microbiome and immunity-related studies, we did not observe the statistical significance of sex differences in our experiments.

**Fig. 7:**
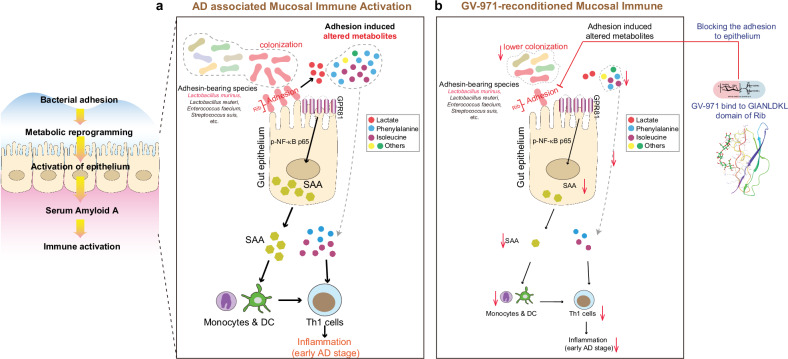
Schematic diagram of how *L.m*. bearing adhesion domain like Rib provokes Th1 cell activation in AD-associated neuroinflammation and the intervention strategy elicited by GV-971. **a** The colonization of *L.m*. via adhesion-related Rib domain initiated metabolic reprogramming including the altered amino acid and lactate. The release of lactate could activate SAA via GPR81-p-NF-κB p65 signaling pathway. SAA then activates monocytes and DCs which subsequently stimulate Th1 cells, mediating AD-associated neuroinflammation. Other species bearing Rib, including *Lactobacillus reuteri*, *Enterococcus faecium*, *Streptococcus suis*, etc., may have similar adhesive potential and Th1 stimulating function in AD development. During this process, phenylalanine, isoleucine, and other amino acids also increase to stimulate Th1 cells as mentioned in our last report. **b** Oral administration of GV-971 inhibits Rib-bearing *L.m*. adhesion and decreases bacteria colonization, suppresses the lactate-GPR81-SAA axis, and inhibits phenylalanine, and isoleucine, which finally reduces Th1 cell activation.

Collectively, this work advanced the understanding of the relationship between changes in gut microbiota and Th1-skewed pro-inflammation of AD. How gut microbiota change occurred in the first place remains unclear. It is possible that genetic alterations, such as those altered in Tg mice, might have an impact on the microbiome composition. Nevertheless, we have discovered, for the first time, that Rib^high^ bacteria VFs are the main mediators behind the attachment of bacteria to gut epithelial cells, leading to disease stage-specific metabolic reprogramming (Fig. 7a). Moreover, we were able to gain a better understanding of how GV-971 harnessed the activation of Th1 cells by specifically targeting Rib^high^ bacteria species crucial for adhering to gut epithelial cells (Fig. 7b). These findings highlight the potential of exogenous sugar molecules as a valuable resource for screening microbiota-centric drugs, particularly due to their ability to disrupt bacteria–host interactions. Our findings pave the way for identifying specific glycosylated mucin-binding proteins like Rib that are associated with disease stages in the future, enabling targeted interventions during the onset and progression of AD.

The limitations of this study include the following. First, the lack of agents to directly modulate the gut epithelial GPR81-SAA axis limited our in vivo investigations. Future studies with ileum epithelial-specific GPR81 or SAA gene knockout mice are essential for deeper exploration. Second, we should include more mice of varying ages and conditions to capture the dynamic patterns of microbiota, molecules, and immune cells over time. Third, our findings did not explore direct evidence linking our results to canonical AD pathology markers like Aβ deposition, potentially due to insufficient experimental time for macroscopic AD pathology to develop. Extending the experimental treatment time in FMT assays to investigate changes in these markers requires further exploration. Lastly, expanding human sample testing is necessary to assess the translational value of Rib and other mucin-binding proteins in AD progression.

## Materials and methods

### Reagents

This experiment required a plethora of resources and reagents. Various antibodies were employed, including the Human Anti-GPCR GPR81 antibody from Abcam (ab106942), Human Recombinant Anti-SAA1 + SAA2 antibody from Abcam (ab207445), Recombinant anti-Actin antibody from Abcam (ab179467), and Phospho-NF-κB p65 (Ser536) Rabbit mAb (3033) from Cell Signaling Technology. The antibodies Dnk pAb to Goat IgG (HRP) from Abcam (ab97110), Goat pAb to Rb IgG (HRP) from Abcam (ab97051), Rabbit anti-SAA antibody from Abcam (ab199030), and Peroxidase AffiniPure Goat Anti-Rabbit IgG (H + L) from JACKSON (111-035-003) were also utilized. Mouse ELISA kit for SAA detection was from Abcam (ab157723). Biolegend provided the Live/Dead marker (423104), Anti-CD16/32 (101320), as well as antibodies for CD3 (152316), CD4 (100406), CD45 (103116), CD11b (101205), CXCR3 (126522), CCR6 (129814), CCR4 (131204, 131214), IFNγ (505836), Foxp3 (320008), IL-4 (504120), Ly6C (128044), and Ly6G (127616). BD supplied antibodies for CD4 (563790) and CD11b (564454).

Bacterial strain *L.m*. and GV-971, featured in the study, was self-reported in the manuscript. QUANTI-Blue solution from InvivoGen (San Diego, CA, USA, rep-qlc2), DL-Sodium Lactate from MERCK (L4263-100ML), 3-chloro-5-hydroxybenzoic acid (3-Cl-5-OHBA) from TGI (#53984-36-4), and 3,5-dihydroxybenzoic acid from Sigma (D110000-100G). Recombinant human Apo-SAA1 from PeproTech, USA (300-53), Recombinant Mouse Serum Amyloid A1 Protein, CF, no BSA from R&D systems (2948-SA-025). Experimental models employed included human peripheral blood mononuclear cells (PBMCs) sourced from MiaoTong-Biology, THP1-Dual cells from InvivoGen (San Diego, CA, USA, thpd-nfis), and Human HT29 cells from ATCC (HTB-38™). Mouse models used were B6SJL-Tg^(APPSwFlLon,PSEN1*M146L*L286V)^6799Vas/Mmjax (5XFAD, Tg6799) from The Jackson Laboratory and C57BL/6 J, WT mice from Shanghai Lingchang Biotechnology. Oligonucleotides used in the study included PCR primers Lac1 (F) and Lac2 (R), along with si*GPR81* #1 and si*GPR81* #2, all from Sangon Biotech. The sequences of these were 5’-AGCAGTAGGGAATCTTCCA-3’, 5’-ATTYCACCGCTACACATG-3’, 5’-CUGCUAGACUCUAUUUCCU(TT)-3’, and 5’-GUUGCAUCAGUGUGGCAAA(TT)-3’, respectively. Various software and algorithms for analysis and interpretation of data included fastp, FLASH, UPARSE, BWA, SOAPdenovo, MetaGene, CD-HIT, and BLASTP for data processing, and Adobe Illustrator CC, Adobe Photoshop CC, GraphPad Prism, and ImageJ for visualization and illustration.

### Mice

5XFAD Tg mice (B6SJL-Tg^(APPSwFlLon,PSEN1*M146L*L286V)^6799Vas/Mmjax (5XFAD, Tg6799)) and C57 WT mice were bred separately after birth. All the mice were maintained in a room at 23 °C under a 12-h light-dark cycle. Mice were randomly allocated to different groups before treatment. For time course analysis of Tg mice, male and female Tg mice were sacrificed at 24-, 33-, and 41-week-age (or 5-, 8- and 10-month-age). Before the mice were sacrificed, their feces were collected in the morning around 10 o’clock for further experiments. The animal experiments were approved by the ethical committee of Shanghai SIPPR-Bk Lab Animal Co., Ltd. (Number: 202001). Male mice were used as recipient mice in FMT assays unless otherwise specified. The numbers and sexes of animals used in other parts of this study are detailed in Figure Legends and Supplementary Table S6.

### Patient selection

All participants provided informed consent, and the study was approved by the ethics committee of Shanghai Sixth People’s Hospital, Shanghai Jiao Tong University School of Medicine under reference number #2019-032. All participants were enrolled in a standardized examination, during which they provided their medical history and information about their disease. They underwent relevant laboratory screenings and received a comprehensive set of neuropsychological assessments and cranial MRI. To be included in the study, participants had to meet certain criteria: they were required to be over 50 years old, have at least 6 years of education, have normal eyesight and hearing, and have no history of alcoholism, drug abuse, head trauma, or other neuropsychiatric diseases such as depression and anxiety that could impact their performance. They also had to have no apparent abnormalities in folic acid, vitamin B12, thyroid function, rapid plasma regains, or treponema pallidum particle agglutination.

For the cognitively normal participants, their inclusion criteria involved being generally healthy at the time of enrollment, as determined by evaluating their medical history and assessing their vital signs. At the time of enrollment, individuals with an active systemic or serious concurrent illness, cognitive impairment, or a history of immunodeficiency were excluded from the study. The criteria for enrolling participants with AD included: (1) meeting the National Institute of Aging and Alzheimer’s Association (NIA-AA) criteria for probable AD^62^; (2) having a Hachinski Ischemia Score of less than 4; (3) identification of a responsible and consistent caregiver; (4) absence of significant diabetes, renal impairment, systemic conditions, psychiatric disorders, seizures, or traumatic brain injuries that could compromise their cognitive or brain functions; (5) significant brain abnormalities on the patient’s T1-weighted MRI; (6) mild-to-moderate AD with a Mini-Mental state Examination (MMSE) score ranging from 11 to 24.

### Isolation and identification of *L.m*. bacterial strain

*L.m*. was isolated from mice fecal samples that were aseptically spread on MRS agar (288210, BD, USA) for isolating. 100 mg fresh or frozen mice fecal samples were first collected and were then diluted in 2 mL sterile anaerobic phosphate-buffered saline (PBS) (pH 7.2; containing *L*-cysteine at 0.1%). The solution was 10-fold serially diluted using the same PBS buffer and then the 10^−4^ dilutions were spread onto an MRS agar plate containing *L*-cysteine at 0.05% and CaCO_3_ at 0.4%. Then, the plate was incubated at 37 °C inside the anaerobic incubator for 48 h. After 48 h, select colonies based on the colony morphology of *Lactobacillus* and the calcium-dissolving zone. Next, we used bacterial PCR to isolate target bacteria from the selected colonies. Amplification was performed in a 50 μL reaction mixture containing 2× PCR buffer for KOD FX, 2 mM dNTPs, 1 μL KOD FX (1.0 U/μL) (KFX-101, TOYOBO, Japan), 10 pmol of each forward primer and reverse primer, and a little number of bacteria clones on the plate. We used *Lactobacillus* group-specific primers, Lac1: 5’-AGCAGTAGGGAATCTTCCA-3’ and Lac2: 5’-ATTYCACCGCTACACATG-3’. PCR was performed using the Bio-Rad (T100, USA) with the following conditions: 94 °C for 2 min; 35 cycles of 98 °C for 10 s, 60 °C for 30 s, and 68 °C for 1 min; and finally, 68 °C for 7 min. The target sequence length is about 340 bp and is identified using 1% agarose gel (111860, Bio-west, USA) electrophoresis at the condition of 120 V/m, 40 min. For the isolated *L.m*., we cultured colonies in MRS broth liquid media at 37 °C under anaerobic conditions for 18–24 h to reach the log-phase growth period. For preservation, we subtracted bacterial-containing MRS media with 50% glycerol (G66258A, GENERAL-REAGENT, Tansole, Taitan, Shanghai, China) and freezed at –80 °C until needed. When needed, prepare working cultures from stock cultures, and propagate twice prior to use by sub-culturing in MRS broth liquid media (288130, BD) supplemented with 0.05% L-cysteine.

### Lactate detection

Lactate was detected as previously described^63^. Mouse feces were completely homogenized in water, and then sonicated on ice for 15 min. Pyridine buffer was prepared by combining 1.8 mL of HCl (12 mol/L), 2.87 mL of pyridine, and 28.7 mL of water (pH 5.0). 25 μL of standard curves and samples were mixed with 50 μL of 1 M O-Benzylhydroxylamine and 50 μL of 1 M 3-(ethyliminomethylideneamino)-*N*, *N*-dimethylpropan-1-amine in the pyridine buffer. After 30 min at room temperature, 300 μL of ethyl acetate was added and the plates were shaken for 20 min centrifuged for 5 min at 1900 rpm, and then 50 μL of organic layer was taken into a 96-well plate. Then the aqueous layer was dried using a stream of nitrogen at 40 °C and reconstituted in 300 μL of methanol/water (50: 50, v/v). The samples were analyzed by a quantitative liquid chromatography with tandem mass spectrometry (LC-MS/MS).

### *L.m*. aggregation assays

The aggregation assays were adapted based on previous studies^64^. Grow *L.m*. bacterial cells for 18 h at 37 °C in MRS broth, harvest cells by centrifugation at 5000× *g* for 15 min, washed twice, and resuspend in phosphate-buffered saline (PBS; pH 7.4) to give viable counts of approximately 10^8^ CFU/mL. Then, cell suspensions (4 mL) were mixed by vortexing for 10 s and aggregation was determined during 120 min of incubation at room temperature. For every hour, 0.1 mL of the upper suspension was transferred to another tube with 3.9 mL of PBS and the absorbance was measured at OD_600_ nm. The aggregation ratio was calculated as 1 − (A_t_/A_0_)*100 where A_t_ represents the absorbance at time t = 0, 15, 30, 60, 120 min and A_0_ the absorbance at t = 0 min.

### *L.m*. adhesion assays, GV-971 treatment, and western blot analysis

The adhesion assays were adapted based on previous studies^40^. Prepare *L.m*. bacterial working cultures using MRS broth under anaerobic conditions, and centrifuge the cultures twice using PBS at 3000× *g*, 4 °C for 5 min at room temperature. Weight the mass of bacterial pellets and treat the pellets with 5 mL PBS per 10–15 mg bacterial pellets, then place the mixture on wet ice or 0 °C for 30 min. After 30 min, collect bacterial pellets at 3000× *g*, 4 °C for 20 min, then resuspend and rinse the pellets using PBS buffer at 3000× *g*, 4 °C for 5 min. Resuspend the pellets using PBS and adjust the concentration of bacteria to 1 × 10^8^ CFU/mL. Prepare HT29 cells and seed them at the cell fusion proportion of about 80%–90% into 12-well plates that are pre-placed with poly-L-lysine (P2636, Sigma, USA) coated glass slices one day before experiment. The coating procedure is as follows: the glass slides (41001112, Assistent, Germany) were sterilized and then put into 12-well plates, added with 1 mL poly-*L*-lysine at 37 °C for 3 h, then washed with distilled water for 3 times. On the experiment day, pipette HT29 medium and carefully rinse the cells twice using PBS. Add 1 mL DMEM medium, and then add 1 mL suspensions containing bacteria. Different concentrations of GV-971 were added simultaneously with bacteria suspension. Carefully mix the culture and place the plates at 37 °C for 2 h. Afterward, pipette the medium, then rinse with PBS three times, fix with methanol (1 mL) for 20 min, rinse with PBS twice, and then move to gram staining (G1060, Solarbio, Beijing, China) and microscopic observation (100× oil lense, 1000× magnification in total). Five views were randomly chosen for each image. The number of *L.m*. bacteria adhering to HT29 cells was calculated on bacterial counts per cell. For western blot analysis, after mixing the culture at 37 °C for 2 h, pipette the medium, rinse with PBS three times, then perform cell lysates with 1× Lysis Buffer^65^, followed by staining with specific protein antibodies and analyzed by western blot analysis. Protein expression levels were quantified with Image Lab software. All experiments were repeated in triplicate.

### Bacterial-HT29 conditioned medium collection for detection of metabolites

We propagated twice prior to use by sub-culturing in MRS broth liquid media (288130, BD) supplemented with 0.05% L-cysteine. Centrifuge the cultures twice using PBS at 3000× *g*, 4 °C for 5 min. Weight the mass of bacterial pellets and treat the pellets with PBS at 5 mL PBS per 10–20 mg bacterial pellets. Resuspend the pellets with MRS broth at 1 mL MRS broth per 20 mg bacterial pellets. The pellets were grown at 1 mL per well in 96-deep well plate. Then, the plate was incubated at 37 °C under inside the anaerobic incubator for 24 h. Rib^high^-*L.m*. was cultured with MRS broth for 24 h following previous passage under anaerobic conditions in the anaerobic chamber. The supernatant was collected at 3000× *g*, 4 °C for 20 min (named MRS + Rib^high^-*L.m*.) and stored at –80 °C upon usage or for immediate co-culture assays.

For co-culture assays, HT29 cells were grown at 500,000 cells per well in a 12-well plate for 24 h until cell adhesion. The next day, we discarded HT29 medium and rinsed cells with PBS two times, then added 1.75 mL DMEM medium mixed with 0.25 mL MRS broth cultured with *L.m*. (MRS + Rib^high^-*L.m*.) for another 48 h under normal cell culture condition at 37 °C in a humidified atmosphere of 5% CO_2_ and 95% air. In GV-971 treatment assays, different concentrations of GV-971 were added together with MRS + Rib^high^-*L.m*. and incubated with HT29 for 48 h. After 48 h, the supernatant was collected for further immediate studies or stored at –80 °C after aliquot, with the supernatant named the 48 h Rib^high^-*L.m*. MRS-HT29 conditioned medium. The supernatants were then subjected to metabolomics analysis. All experiments were repeated in triplicate.

### Mouse fecal sample collection

All fecal samples were collected in the morning around 10 o’clock. Before collecting, label 1.5 mL or 2 mL cryo-vials as needed. Put one mouse in a separate clean box with no bedding and allow it to defecate naturally without disturbance. Once the mouse had defecated 3–4 fecal pellets, it was removed from the collection cage and the pellets were immediately collected and put into the vial (maximum 4 pellets/vial). Place the vial with fecal sample on dry ice until all samples have been collected and are ready for storage. Once all samples are collected, vials were removed from ice and the samples were stored at –80 °C or until ready for shipping. All shipping procedures were under dry ice conditions to keep fecal samples frozen.

### ABX treatments

Mice were treated by adding an antibiotic solution (ABX) containing ampicillin (0.1 mg/mL, final concentration in drinking water), streptomycin (0.5 mg/mL, final concentration in drinking water), and colistin (0.1 mg/mL, final concentration in drinking water) (Sigma) to sterile drinking water. Solutions and bottles were changed 3 times and once weekly, respectively. The antibiotic activity was confirmed by 16S rRNA-seq. The duration of ABX treatment was slightly different based on the experimental settings. In the context of fecal microbial transplantation experiments, mice received 3 days of ABX before undergoing fecal microbial transplantation the next day by oral gavage using animal feeding needles.

### FMT and *L.m*. oral gavage experiments

FMT was performed by thawing fecal material. Then, 200 μL of the suspension was transferred by oral gavage into each ABX pre-treated recipient. Different recipient mice according to experiment settings were first treated with an ABX cocktail in drinking water for 3 days, and then 40 mg of the mixed stool suspended in PBS was inserted by gavage into each mouse 3 times with a 2-day break in between. For *L.m*. oral gavage, 200 μL of bacterial suspension (10^9^ CFU/mL) was transplanted to each recipient mouse by oral gavage 3 times with a 2-day break in between. The mice were sacrificed 3 days later and used for different analyses. For human microbiota transplant assays, human fecal samples were collected from Shanghai Mental Health Centre. All participants signed informed consent.

### IHC

The following steps were performed by Shanghai Zuocheng Bio. Briefly, first, the intestine samples were sliced (LEICA, Germany, RM2235) and paraffin-embedded (KD-BMII, Zhejiang Jinhua Kedi Instrumental Equipment Co., Ltd.). The slices were dewaxed and hydrated using ethanol and dimethylbenzene as a regular procedure. Then the sections were submitted to heat-induced epitope retrieval with EDTA buffer (pH 9.0) under high pressure (80 Kpa) for 5 min. Endogenous peroxidase activity was blocked with 3% (v/v) hydrogen peroxide using methanol for 10 min. After washed using PBS buffer for 5 min, three times, sections were incubated with blocking buffer (Normal Goat Serum. JACKSON, 005-000-121) for 30 min. Then the sections were incubated with primary antibody overnight at 4 °C. Intestine sample sections were stained with rabbit anti-SAA antibody (1:50, ab199030, Abcam). The next day, sections were washed at room temperature using PBS buffer for 5 min, three times, and then were incubated with a second antibody (Peroxidase AffiniPure Goat Anti-Rabbit IgG (H + L), JACKSON, 111-035-003) for 30 min at room temperature. After washing again using PBS buffer for 5 min, three times, the sections were stained by DAB (Sigma, D8001). Then the stained slices were washed with water for 15 min, re-stained using hematoxylin (ThermoFisher Scientific, 7211), dehydrated using gradient concentration of ethanol, transparented by dimethyl benzene, and then sealed with neutral balsam (Sinopharm Chemical Reagent Co., Ltd, Shanghai, China). Stained slices were automatically scanned by a high-throughput bright field scanner (NanoZoomer S210, Hamamatsu, Japan), and images were obtained by NDP.scan 3.2 software (Hamamatsu).

### In vivo immune test and flow cytometry

Mice were anesthetized, blood samples were collected into EDTA-containing tubes, and red blood cells were removed using 1× red blood lysis buffer. Before tissue collection, the brains were perfused with ice-cold PBS to avoid sampling the circulating blood immune cells, and the brains were removed, chopped into pieces, and dissected according to the introduction of the Adult Brain Dissociation Kit (130-107-677, Miltenyi Biotech) using the gentleMACS dissociator (Miltenyi Biotech). The brain homogenate was filtered through a 70-μm cell strainer and centrifuged at 300× *g* for 5 min at 4 °C. The cell pellet was resuspended in cold PBS buffer and centrifuged again at 300× *g* for 5 min at 4 °C. All samples were counted, labeled with a Live/Dead kit for 30 min, and then centrifuged at 500× *g* for 3 min at 4 °C. The cells were resuspended in 100 μL PBS buffer, blocked with anti-CD16/32 (101320, Biolegend) for 10 min, and incubated with the antibody according to the manufacturers’ protocols at 4 °C for 30 min. The following antibodies were used in the FACS analysis: Live/Dead (423104, Biolegend), CD45(30-F11)-APC-Cy7(103116, Biolegend), CD11b(M1/70)-FITC (101205, Biolegend), CD11b(M1/70)- BB515 (564454, BD), Ly-6G(1A8)-PerCP/Cy5.5 (127616, Biolegend), Ly-6C(HK1.4)-PE-Dazzle 594 (128044, Biolegend), CD3(500A2)-AF700 (152316, Biolegend), CD4(GK1.5)-FITC (100406, Biolegend), CD4 (GK1.5)-BUV395 (563790, BD), CXCR3(CXCR3-173)-BV421 (126522, Biolegend), CCR6(29-2L17)-APC (129814, Biolegend), CCR4(2G12)-PE (131204, Biolegend), CCR4(2G12)- PE/CY7 (131214, Biolegend). Cells were added to 500 μL PBS buffer, centrifuged at 500× *g* for 3 min at 4 °C, and resuspended in 200 μL running buffer. Relevant negative control, Fluorescence Minus One (FMO) control and each fluorescence compensation sample were used to adjust fluorescence compensation and identify the populations of interest. Cells were acquired on a BD Aria III cytometer, and data were analyzed using FlowJo 10.7 software.

### In vitro immune-related assays and flow cytometry

Spleen samples were collected from 12-month-old C57BL/6 male mice. The spleens were homogenized and filtered through a 70-μm cell strainer. The Cell suspension was centrifuged at 500× *g* for 5 min at 4 °C, and red blood cells were removed using 1× red blood lysis buffer. A total of 5 × 10^5^ cells/well in 0.2 mL of RPMI-1640 medium were plated in a 96-well plate.

After incubation at 37 °C in 5% CO_2_ for 20 h, cells were stimulated with Cell activation Cocktail (00-4975-03, ThermoFisher Scientific) in 5% CO_2_ at 37 °C for 4 h, washed twice with PBS, and labeled with zombie yellow (423104, Biolegend) to exclude dead cells. Nonspecific binding of immunoglobulin to the Fc receptors was blocked with anti-CD16/32 (101320, Biolegend). Cell staining was conducted according to the manufacturers’ protocols. The following antibodies were used in the FACS analysis: CD4(GK1.5)-FITC (100406, Biolegend), IFNγ(XMG1.2)-BV711 (505836, Biolegend), Foxp3 (150D)-PE (320008, Biolegend), IL-4 (11B11)-BV421 (504120, Biolegend), CD11b(M1/70)-BB515 (564454, BD), Ly6C(HK1.4)- PE/Dazzle594 (128044, Biolegend), Ly6G(1A8)- PerCP/Cy5.5 (127616, Biolegend). After staining, cells were resuspended in 200 μL running buffer. Relevant negative control, FMO control, and each fluorescence compensation sample were used to adjust fluorescence compensation and identify the populations of interest. Cells were acquired on a BD LSRFortessa cytometer, and data were analyzed using FlowJo software.

Gating strategy: FSC-A/FSC-H for single cells, Zombie yellow BV510 for live cells. CD45^+^CD11b^+^Ly6G^–^Ly6C^+^ to gate Monocytes. For in vivo experiment, CD45^+^CD4^+^CXCR3^+^CCR6^–^ to gate Th1 cells, For in vitro experiment, CD4^+^IFNγ^+^ΙL-4^–^ for gating Th1 cells.

### hSAA treatment of THP1-Dual cells

THP1-Dual cells were plated into 24-well plates at a density of 5 × 10^5^ viable cells in 1 mL of medium per plate and incubated with different concentrations of hSAA for 24 h. Then, the culture supernatants were measured for the activation of NF-κB reporter and cytokines. Briefly, for NF-κB reporter assay, 20 μL of supernatant and 180 μL of QUANTI-Blue detection reagent were incubated at 37 °C for 1 h in a humidified atmosphere of 5% CO_2_ and 95% air, and subsequently the absorbance was measured at 650 nm by a SpectraMAX plus 384 (Molecular Devices, CA). The fold change of NF-κB reporter was calculated relative to vehicle control. For cytokines assay, 50 μL of supernatant was measured by Bio-Plex Pro Human Cytokine Grp I Panel 27-plex kit (M500KCAF0Y, Bio-Rad) according to the manufacture’s protocol by using the Bio-Plex MAGPIX System. This kit can quantify the concentration of 27 cytokines including IL-6, TNFα, IL-12, and IFN-γ.

### Co-culture of hSAA-induced THP1-Dual and CD4^+^ T cells

THP1-Dual cells were plated into 24-well plates at a density of 5 × 10^5^ viable cells in 1 mL of medium per well and incubated with vehicle or hSAA for 24 h. CD4^+^ T cells was isolated from human PBMC by using the EasySep™ Human CD4^+^ T Cell Isolation Kit (Stem Cell Technologies) according to the manufacture’s protocol. For Th1 cell differentiation assay, vehicle, or hSAA-induced THP1-Dual cells (5 × 10^5^ per well) were co-cultured with isolated CD4^+^ T cells (1:1) for 3 days, and the percentage of Th1 cell differentiation was detected by the flow cytometer using the biomarker CD4 and IFN-γ. For cytokines assay, vehicle or hSAA-induced (0.8 µg/mL, 24 h) THP1-Dual cells (5 × 10^5^ per well) were co-cultured with CD4^+^ T cells (1:1) for 3 days, the secretion of 27 cytokines in culture supernatants was determined by Bio-Plex Pro Human Cytokine Grp I Panel 27-plex kit (M500KCAF0Y, Bio-Rad) as shown above.

### Induction of mouse BMDCs

Bone marrow cells were isolated from the back leg bone of C57 mice. Briefly, in a laminar flow hood using sterile utensils, cut both ends of the bone of C57 mice with scissors as close to the joints as possible. Fill a syringe with ice-cold RPMI complete media, insert the syringe needle into the bone, and flush out the bone marrow into a centrifuge tube on ice. Red blood cell lysis buffer (1×) was used to lyse cells for 3 min. Centrifuge the lysis twice in RPMI-1640 medium at 1500 rpm for 5 min.

For BMDC induction, dilute the cells into 1 mL of RPMI-1640 medium with 20 ng/mL GM-CSF and 10 ng/mL IL-4. Then place in 24-well plates at a density of 1 × 10^6^ viable cells per well and incubated in a 37 °C incubator with 5% CO_2_. Add 1 mL of RPMI-1640 medium with 20 ng/mL GM-CSF and 10 ng/mL IL-4 on day 3. Remove half of the media on day 6, and briefly centrifuge the removed media to cell pellet and resuspended in 1 mL of fresh RPMI-1640 medium with 20 ng/mL GM-CSF and 10 ng/mL IL-4 and put back to the well. Mature BMDCs were harvested on day 8.

### mSAA treatment of BMDCs

BMDCs were plated into 24-well plates at a density of 5 × 10^4^ viable cells in 1 mL of medium per plate and incubated with 10 μg/mL of mSAA for 24 h. For cytokines assay, 50 μL of supernatant was measured by Bio-Plex Pro Mouse Cytokine Grp I Panel 23-plex (M60009RDPD, Bio-Rad) according to the manufacture’s protocol by using the Bio-Plex MAGPIX System. This kit can quantify the concentration of 23 cytokines as indicated by using each of its standard curves.

### Co-culture of mSAA-induced BMDC or BMDM and CD4^+^ T cells

BMDCs were plated into a 24-well plates at a density of 5 × 10^4^ viable cells in 1 mL of medium per plate and incubated with vehicle (1% PBS) or mSAA (10 µg/mL) for 24 h. CD4^+^ T cells was isolated from mouse spleen by using the EasySep™ Mouse CD4^+^ T Cell Isolation Kit (Stemcell Technologies). For cytokines assay, vehicle or mSAA-induced BMDC (5 × 10^4^ per well) were co-cultured with CD4^+^ T cells (1:10) for 3 days, and the secretion of cytokines in culture supernatants were determined by Bio-Plex Pro Mouse Cytokine Grp I Panel 23-plex (M60009RDPD, Bio-Rad) as shown above.

### Fecal sample DNA extraction, PCR amplification and 16S rRNA-seq

All fecal samples were frozen at −80 °C before DNA extraction and analysis. The following steps were conducted by Majorbio Bio-Pharm Technology Co., Ltd. (Shanghai, China). Microbial DNA was extracted from fecal samples using the E.Z.N.A.® Soil DNA Kit (Omega Bio-Tek, Norcross, GA, U.S.) according to the manufacturer’s protocols. The final DNA concentration and purification were determined by a NanoDrop 2000 UV–vis spectrophotometer (ThermoFisher Scientific), and DNA quality was checked by 1% agarose gel electrophoresis. The V3–V4 hypervariable regions of the bacterial 16 S rRNA gene were amplified with primers 338 F (5’-ACTCCTACGGGAGGCAGCAG-3’) and 806 R (5’-GGACTACHVGGGTWTCTAAT-3’) by a thermocycler PCR system (GeneAmp 9700, ABI). PCR reactions were conducted using the following program: 3 min of denaturation at 95 °C, 27 cycles of 30 s at 95 °C, 30 s for annealing at 55 °C, and 45 s for elongation at 72 °C, and a final extension at 72 °C for 10 min. PCR reactions were performed in triplicate in a 20 μL mixture containing 4 μL of 5× FastPfu Buffer, 2 μL of 2.5 mmol/L dNTPs, 0.8 μL of each primer (5 μmol/L), 0.4 μL of FastPfu Polymerase and 10 ng of template DNA. The resulting PCR products were extracted from a 2% agarose gel and further purified using the AxyPrep DNA Gel Extraction Kit (Axygen Biosciences) and quantified using QuantiFluor™-ST (Promega) according to the manufacturer’s protocol. Purified amplicons were pooled in equimolar and paired end sequenced (2 × 300) on an Illumina MiSeq platform (Illumina).

### Processing of 16S rRNA-seq data

The following steps were conducted by Majorbio Bio-Pharm Technology Co., Ltd. Raw fastq files were demultiplexed, quality filtered by fastp (https://github.com/OpenGene/fastp, version 0.20.0), and merged by FLASH (version 1.2.7) based on the following criteria: (i) the reads were truncated at any site that received an average quality score < 20 over a 50 bp sliding window; (ii) The primers were exactly matched, allowing a 2-nucleotide mismatch, and reads containing ambiguous bases were removed; (iii) Sequences with overlaps of longer than 10 bp were merged according to their overlap sequence. Operational taxonomic units (OTUs) were clustered with a 97% similarity cut-off using UPARSE (version7.1 http://drive5.com/uparse/), and chimeric sequences were identified and removed using UCHIME. The taxonomy of each OTU representative sequence was analyzed by the RDP Classifier algorithm (http://rdp.cme.msu.edu/) against the Silva 16S rRNA database (silva 132/16s bacteria) using a confidence threshold of 70%.

### Fecal sample DNA extraction, library construction, and metagenomics sequencing

All fecal samples were frozen at –80 °C before DNA extraction and analysis. The following steps were conducted by Majorbio Bio-Pharm Technology Co., Ltd. Microbial DNA was extracted from fecal samples using the E.Z.N.A.® Soil DNA Kit (Omega Bio-Tek) according to the manufacturer’s protocols. The DNA concentration and purity were quantified with TBS-380 and NanoDrop2000, respectively. DNA quality was examined using the 1% agarose gel electrophoresis system. DNA was fragmented to an average size of about 400 bp using Covaris M220 (Gene Company Limited) for paired-end library construction. The paired-end library was prepared by using TruSeqTM DNA Sample Prep Kit (Illumina). Adapters containing the full complement of sequencing primer hybridization sites were ligated to the Blunt-end fragments. Paired-end sequencing was performed on Illumina NovaSeq platform (Illumina) at Majorbio Bio-Pharm Technology Co., Ltd. using NovaSeq Reagent Kits according to the manufacturer’s instructions (www.illumina.com).

### Metagenomics sequence quality control and genome assembly

The following steps were conducted by Majorbio Bio-Pharm Technology Co., Ltd. The paired-end Illumina reads were trimmed of adaptors, and low-quality reads (length < 50 bp or with a quality value < 20 or having N bases) were removed by fastp (https://github.com/OpenGene/fastp, version 0.20.0). Reads were aligned to the mouse genome (https://www.ncbi.nlm.nih.gov/genome/52) by BWA (http://bio-bwa.sourceforge.net) and any hit associated with the reads and their mated reads were removed. Metagenomics data were assembled using SOAPdenovo (http://soap.genomics.org.cn, Version 1.06, which makes use of succinct de Bruijn graphs. Contigs with a length being or over 300 bp were selected as the final assembling result, and then the contigs were used for further gene prediction and annotation.

### Metagenomics gene prediction, taxonomy, functional annotation

The following steps were conducted by Majorbio Bio-Pharm Technology Co., Ltd.

#### Gene prediction and catalog

MetaGene (http://metagene.cb.k.u-tokyo.ac.jp/) was used for open reading frame (ORF) prediction. Removing redundancy was done with CD-HIT (http://www.bioinformatics.org/cd-hit/, version 4.6.1), and sequences with 95% identity and 90% coverage were considered redundant. The predicted ORFs with length being or over 100 bp were retrieved and translated into amino acid sequences using the NCBI translation table (http://www.ncbi.nlm.nih.gov/Taxonomy/taxonomyhome.html/index.cgi?chapter=tgencodes#SG1). Taxonomy identification was performed by BLASTP search (http://blast.ncbi. nlm.nih.gov/Blast.cgi, BLAST version 2.2.28+) with Evalue < 1 × 10^−5^ against the non-redundant (NR) database.

#### COG, KEGG, CAZyme, and VFDB annotations

The catalog, comprised of non-redundant coding sequence correspondent protein sequences, was annotated to achieve the COGs, based on the evolutionary genealogy of genes: Non-supervised Orthologous Groups (eggNOG), using BLASTP (BLAST version 2.2.28+) with Evalue < 1 × 10^–5^. This catalog was also annotated to inform the metabolic pathway based on the Kyoto Encyclopedia of Genes and Genomes (KEGG), using BLASTP (BLAST version 2.2.28+) with Evalue < 1 × 10^–5^. These catalogs were searched in the carbohydrate-active enzyme (CAZyme) database (http://www.cazy.org/, version 5.0), which offers functional classification in categories and families to annotate the CAZyme family. Virulent factor annotations were conducted using BLASTP search (Version 2.2.28+) against VFDB database (http://www.mgc.ac.cn/VFs/) with E value < 1 × 10^–5^.

### Chain-specific prokaryotic single bacteria transcriptomics sequencing

The experiment used the TruSeqTM Stranded Total RNA Library Prep Kit for library construction. In the synthesis of the second cDNA strand, dUTP was used to replace dTTP in the dNTPs reagent, resulting in the second cDNA strand containing the bases A/U/C/G. Before PCR amplification, the second cDNA strand was digested with UNG enzyme, so that the library contained only the first strand of cDNA. **A** Total RNA Extraction. Total RNA was extracted from tissue samples, and its concentration and purity were measured using Nanodrop2000. RNA integrity was checked by agarose gel electrophoresis, and the RIN value was determined by Agilent2100. A single library construction requires a total RNA amount of 2 µg, concentration ≥100 ng/µL, and OD260/280 between 1.8–2.2. **B** Removal of rRNA. Unlike eukaryotic mRNA, which has a ployA tail at the 3’ end, prokaryotes cannot use Oligo dT to pair with ployA for A–T base pairing, hence mRNA is separated from total RNA. Generally, rRNA is removed for transcriptome analysis. **C** Fragmentation of mRNA. The Illumina platform sequences short sequence fragments. The enriched mRNA is the complete RNA sequence with an average length of several kb, so it needs to be randomly broken down. Adding fragmentation buffer, the mRNA can be randomly broken into small fragments of about 200 bp. **D** Reverse Transcription of cDNA. Under the action of reverse transcriptase, using random primers and mRNA as a template, single-stranded cDNA is reverse transcribed. During the synthesis of the second strand, dUTP replaces dTTP in the dNTPs reagent, resulting in the second cDNA strand containing bases A/U/C/G. **E** Adaptor Ligation. The double-stranded cDNA structure has sticky ends. End Repair Mix is added to make blunt ends, and then an A base is added to the 3’ end for connecting the Y-shaped adapter. **F** Digesting of the second cDNA strand with UNG Enzyme. Before PCR amplification, the second cDNA strand is digested with UNG enzyme, so that the library contains only the first strand of cDNA. **G** Sequencing on Illumina Hiseq. Library enrichment, PCR amplification for 15 cycles; Quantification with TBS380 (Picogreen), mixed for sequencing in proportion to the data; Bridge PCR amplification on cBot to generate clusters; Sequencing on the Illumina Hiseq platform, performing 2*150 bp/300 bp sequencing. Data quality control of raw reads: Remove adapter sequences from the reads; Trim and remove bases at the 5’ end that are not A, G, C, or T; Trim the ends of reads with low sequencing quality (sequencing quality value less than Q20); Remove reads where the proportion of bases containing N reaches 10%; Discard small fragments less than 25 bp in length after adapter removal and quality trimming.

### Untargeted metabolomics metabolites sample preparation

Samples stored at –80 °C were taken out and thawed at room temperature. The following steps were conducted by Majorbio Bio-Pharm Technology Co., Ltd. 50 mg samples were used for the experiment. 400 μL methanol-water (4:1, v/v) were also added to homogenize the sample using a homogenizer for 10 s. The solution was ultrasonically extracted on ice for 10 min and stayed at –20 °C for 30 min, then centrifuged for 15 min at 13,000 rpm at 4 °C. 200 μL supernatant was used for LC-MS analysis. Quality Control (QC) sample was prepared by mixing aliquots of all samples to make a pooled sample and then analyzed using the same method with the analytic samples. The QCs were injected at regular intervals (every 10 samples) throughout the analytical run to provide a set of data from which repeatability can be assessed.

### Untargeted metabolomics LC/MS analysis parameters

The following steps were conducted by Majorbio Bio-Pharm Technology Co., Ltd. LC-MS was performed on AB Sciex TripleTOF 5600^TM^ mass spectrometer system (AB SCIEX). LC Conditions: Column: Acquity BEH C18 column (100 mm × 2.1 mm, i.d., 1.7 µm; Waters). Solvent: The column was maintained at 40 °C and separation was achieved using the following gradient: 5% B–20% B over 0–3 min, 20% B–95% B over 3–9 min, 95% B–95% B over 9–13.0 min; 95% B–5% B over 13.0–13.1 min, and 13.1–16 min holding at 5% B at a flow rate of 0.40 mL/min, where B is acetonitrile: isopropanol 1:1 (0.1% (v/v) formic acid) and A is aqueous formic acid (0.1% (v/v) formic acid). The injection volume was 20 μL. The mass spectrometric data was collected using an AB Sciex TripleTOF 5600TM mass spectrometer system equipped with an electrospray ionization (ESI) source operating in either positive or negative ion mode with a capillary voltage of 5000 V or –4000 V, respectively, sample cone, 40 V, collision energy 5 eV. The source temperature was set at 500 °C, with a desolvation gas flow of 45 L/h. Centroid data was collected from 50 to 1000 m/z with a 30,000 resolution.

### Amino acid detection

A set of amino acid standard mixture solutions was prepared at a concentration range of 100–2000 μmol/L. A portion of 10 μL of each standard mixture solution or plasma sample was pipetted into the bottom of a tube, and then 70 μL sodium borate buffer (200 mmol/L at pH 8.8) was added. After 20 μL of 6-aminoquinolyl-N-hydroxysuccinimidyl carbamate (AQC) (4 mg/mL in acetonitrile) was added, the tube was closed and heated for 10 min at 55 °C to form AQC–amino acid. The solution was then cooled down to room temperature and a 2 μL portion of each solution was injected into the UPLC-ESI-MS system for amino acid analysis without further purification.

### RNA-seq

RNA-seq was performed by the Beijing Genomics Institute. RNA was extracted using TRIzol Reagent (ThermoFisher Scientific) and analyzed using an Agilent 2100 Bioanalyzer. Sequencing was performed using an Illumina HiSeq system. The sequences were aligned to the human genome (hg38).

### Bioinformatics analysis

PCA is a method that simplifies the complexity of high-dimensional data while retaining trends and patterns. PCA reduces data by geometrically projecting them onto lower dimensions called principal components (PCs), to find the best summary of the data using a limited number of PCs. PCoA is the modified version of PCA analysis, in which different distance metrics (e.g., Bray–Curtis distances) can be used, compared to the Euclidean distances that is solely used in PCA. Both PCA and PCoA analysis used in this study were initially performed at Majorbio I-Sanger Cloud platform (www.i-sanger.com) and further in R and Adobe Illustrator for better presentation purposes such as shape, color, and font. Pathway analysis and biological function enrichment analysis were performed using the KEGG. RNA-seq data were analyzed using the R package “DOSE”, “GO.db”, “GSEABase” and “ClusterProfiler”. Only pathways with FDR-corrected *P*-value < 0.05 were represented. Other bioinformatics analyses were conducted using the online platform of the Majorbio I-Sanger Cloud (www.i-sanger.com).

### Statistical analyses

Most of the statistical analyses were performed using the online platform of the Majorbio I-Sanger Cloud (www.i-sanger.com), GraphPad Prism, or Microsoft Excel 2010 for *t*-test. For significantly changing bacteria lists, we used the Wilcoxon rank-sum test, and the *P* value was based on a two-tailed test with FDR corrected, the significant level was set to 0.05, and the 0.95 confidence intervals were calculated through the bootstrap algorithm. For more than two group comparisons, one-way ANOVA was performed on the i-sanger platform. Data with error bars are represented as the mean ± SD or mean ± SEM based on different experiment settings and are explained under each Figure legend when applicable. *P* < 0.05 was considered statistically significant. For image quantification, images were analyzed by ImageJ v1.8.0.

## Supplementary information

Supplementary Information

Supplementary Table S1. Annotation details of contigs related to Rib protein from metagenomics

Supplementary Table S2. Details of differences in Genomic Islands, prophages, Insertion Sequences, CAZyms and smBGCs of WT and Tg Lactobacillus murinus genomes

Supplementary Table S3. Differences of Rib genomic structure on WT and Tg lactobacillus murinus genomes

Supplementary Table S4. Differences of bacterial RNA expression profiles of WT and Tg lactobacillus murinus

Supplementary Table S5. Significant metabolites in the 24 h MRS culture medium of Tg lactobacillus murinus

Supplementary Table S6. Animal numbers and sexes used in this study

## Data Availability

The 16S rRNA and metagenomics sequencing raw files can be found in the NCBI SRA database with the accession numbers PRJNA1034236 and PRJNA1036228. The raw sequencing files of the single WT- and Tg-*L.m*. RNA-seq were deposited in the GEO database with the accession number GSE247212. The complete genomic maps for WT- and Tg-*L.m*. were deposited in NCBI SRA with the accession numbers PRJNA1036701 and PRJNA1036703. Other datasets generated or analyzed during this study are included in the published article. Datasets not deposited but those supporting the current study are available from Lead Contact upon reasonable request. This study did not generate new codes nor new unique reagents. Mouse and reagents used in this study will be made available upon reasonable request following approval by an internal review board and require a completed Materials Transfer Agreement. All other data are available in the main text or the Supplementary materials.
