# Supplementary material for: Sodium oligomannate disrupts the adherence of Ribhigh bacteria to gut epithelia to block SAA-triggered Th1 inflammation in 5XFAD transgenic mice
Source: Cell Discov. 2024 Nov 19;10:115. doi: 10.1038/s41421-024-00725-5 (PMC11573985; doi:10.1038/s41421-024-00725-5)

Supplementary Figure Legends

Supplementary Fig. S1: 16S rRNA sequencing reveals diversity, relative abundance, and predicted phenotype variations of gut microbiota of Wild-type (WT) and 5XFAD transgenic (Tg) mice along with aging preceding cognition impairment.

a-b. InverseSimpson community diversity **(a)** and Chao richness **(b)** of the gut faecal microbiota of WT and Tg mice at week age 24 (w24), 33 (w33) and 41 (w41) (n = 4-10). Box plots represent median (the horizontal line within boxes) and the 75th and 25th percentiles (the top and bottom of each box, respectively). The upper and lower whiskers represent 1.5x the interquartile range (IQR) from the top and bottom of the box, respectively. Statistical significance was determined using two-way ANOVA. a) Statistical significance was determined using two-way ANOVA. Effects of Time and Genotype were significant (Time: $P < 0.01$; Genotype: $P < 0.001$). Post-hoc analysis for single experimental conditions using Tukey's Honest Significant Difference (HSD) test: w33_WT versus w33_Tg: *, adjusted p-value = 0.0211451; w41_WT versus w41_Tg: ***, adjusted p-value = 0.0001661; w24_Tg versus w41_Tg: **, adjusted p-value = 0.0010171. b) Statistical significance was determined using two-way ANOVA. Effects of Time and Genotype were significant (Time: $P < 0.001$; Genotype: $P < 0.001$). Post-hoc analysis for single experimental conditions using Tukey's Honest Significant Difference (HSD) test: w24_WT versus w24_Tg: **, adjusted p-value = 0.0060365.

c-d. Changes in the mean relative abundance of gut faecal microbiota at the phylum **(c)** and family **(d)** level of WT and Tg mice at week age 24, 33 and 41.

e-f. Changes in the relative abundance of the predicted facultative anaerobic **(e)** and gram positive **(f)** phenotype faecal microbiota from WT and Tg mice at week age 24, 33 and 41 (n = 4-10) using the BugBase microbial phenotype prediction tool. Data represent as mean ± standard deviation (sd). *$P < 0.05$, by pairwise Mann-Whitney-Wilcoxon Tests. ns, not significant.

g. Comparison of the relative abundance changes (represented as numbers in log fold changes) of specific gut microbiota at the species level in fecal microbiota transplantation (FMT) and antibiotics (ABX) experiments, revealed by metagenomics. For FMT, data represents as the log fold changes of the relative abundance of gut fecal microbiota of the 7-month-old recipient Tg mice that received 14-month-old Tg feces (+Tg feces) versus 14-month-old WT feces (+WT feces). For ABX, data represents as the log fold changes of the relative abundance of gut fecal microbiota of the antibiotics (ABX)-treated 7-month-old Tg mice (Tg+ABX) versus 7-month-old Tg. The middle colour stream plot labels the name of changed species mentioned in Fig. 1d-e, with the ribbons connecting the same species. Red dot, up-regulated; blue dot, down-regulated.

Supplementary Fig. S2: Metabolic changes of WT and Tg mice faeces

a. Mean relative abundance changes of faecal microbiota glycolysis and gluconeogenesis pathways, and fatty acid pathways, and phenylalanine metabolism of WT and Tg mice faeces at week age 24 (w24), 33 (w33) and 41 (w41). * $P < 0.05$, ** $P < 0.01$, by one-way ANOVA.

b-c. Principal component analysis (PCA) of faecal metabolites detected in the positive **(b)** and negative **(c)** ion mode of WT and Tg mice at week age 41 (w41) detected by untargeted metabolomics. Ellipses cover 95% of the data for each group of gut faecal metabolites (n = 4).

Supplementary Fig. S3: SAA expression and functional enrichment results of the ileum tissue RNA sequencing from the recipient mice in FMT assays.

a-c. Expression levels of *Saa1* **(a)**, *Saa2* **(b)** and *Saa3* **(c)** in the ileum tissue of the 7-month-old Tg mice that received 41-week-old WT or Tg faecal samples.

d-g. KEGG (Kyoto Encyclopedia of Genes and Genomes) gene enrichment analysis of the significant up- **(d-f)** or down-regulated **(g)** genes in the ileum tissue of the 7-month-old Tg mice that received Tg faecal samples compared to those receiving WT feces. BP, biological process; MF, molecular function; CC, cellular components; APC, antigen presenting cell. All recipient mice in FMT are male mice.

Supplementary Fig. S4: SAA stimulation on immune cells

a. THP1-Dual cells were incubated with serial concentrations of human SAA (hSAA) from 10 μg/ml to 0.05 μg/ml for 24 hours, the activation fold change of NF-κB reporter was determined and showed as indicated, also see Materials and Methods. Data represent as mean ± standard error of mean (SEM), *** $P < 0.001$ by One-way ANOVA, $n = 3$.

b-c. The secretion of IL-6 and TNF-α **(b)** and IL-12 and IFN-γ **(c)** were shown individually. THP1-Dual cells were treated with 0.8 μg/ml human SAA (hSAA) for 24 hours, the secretion of cytokines in the culture supernatant were determined by cytokine array. *** $P < 0.001$ by Student's *t*-test, $n = 3$.

d. Ability of hSAA to induce Th1 cells on naïve $CD4^+$ T cells without THP1-Dual co-culture. hSAA (10 μg/ml, 24 hours) was added to naïve $CD4^+$ T cells for 3 days, and the frequency of Th1 cells was detected by the flow cytometer. Data are mean ± standard error of mean (SEM). ns, not significant.

Supplementary Fig. S5

a. Changes in the relative abundances of the virulent factor Rib domain of WT and Tg mice feces at week age 24, 33 and 41 (n = 4-10). Abundances of Rib in Tg mice feces but not in WT increased along AD progression. Data represent as mean ± standard deviation (sd). Red asterisk and line, the significance level between week age 41 Tg and week age 24 Tg; black asterisk and line, the significance level between WT and Tg at each time point. *$P < 0.05$, ***$P < 0.001$, by Student's *t*-test.

b. Genomic map of the Rib paralogs on the chromosome of WT *Lactobacillus murinus* sequenced by bacteria complete map sequencing plotted using Circos software. Outer ring to inner ring signifies information of genome size (number in mega base pair, Mbp), genomic information of coding sequence area (CDS) on positive chain and negative chain (color labels show different COG function of CDS), location of Rib (purple line), location of genomic island (GI, in yellow line), location of insertion sequence (IS, in black line), GC% contents (outer red or inner blue bar indicate that the GC% of this area is higher or lower than the average GC content of the whole genome. The higher the bar, the larger the differences) and GC-Skew value (or (G-C)/(G+C) value used to define leading and lagging strands).

c. Different numbers of defensive and offensive virulence factors from WT and Tg *Lactobacillus murinus.*

d. Lactate levels within the bacteria in the 24-hour cultured Rib^{low}-*L.m* and Rib^{high}-*L.m.* *$P < 0.05$ by Student's *t*-test.

e-f. Changes of phenylalanine **(e)** and isoleucine **(f)** in the 48 hours Rib^{high}-*L.m.* MRS-HT29 conditioned (cond.) medium (+Rib^{high}-*L.m.*) versus the MRS-HT29 cond. medium without *Lactobacillus murinus* culture (Con). **$P < 0.01$, by Student's *t*-test.

g. Effect of 50 mM 3-Cl-5-OHBA, the lactate receptor GPR81 agonist, on the protein level of GPR81, p-NF-kB p65 and SAA in HT29 cells.

h. Effect of transient knockdown of GPR81 (small interference RNA, or siGPR81) on the protein level of GPR81, p-NF-kB p65 and SAA in HT29 cells that stimulated by 2 hours' treatment of 10 mM 3-5-DHBA. All siRNAs were added to HT29 cells with RNAimax 6 hours before stimulating by 3-5-DHBA. After 2 hours' stimulation, HT29 cells were subjected to western blotting with the indicated antibodies.

i. Relative abundances of Rib-containing bacteria in health control (HC) and AD patients.

Supplementary Fig. S6: Other Rib structures and docking results with GV-971

a. Sequence alignment of Rib_gene0115, Rib_gene0116, Rib_gene1738, Rib_gene1850, Rib_gene2116 and Rib_gene2117 located in the chromosome of Tg *lactobacillus murinus* sequenced by *de novo* complete map bacteria genome sequencing. Created by CLUSTALW (https://www.genome.jp/tools-bin/clustalw) and ESPript 3.0 (https://espript.ibcp.fr/ESPript/cgi-bin/ESPript.cgi) using default parameters. Black line indicates representative domain of the GIAN* repeats.

b. Example of optimal conformations of GV-971.

c-f. Static and dynamic binding of Tg derived, Rib^{high}-*Lactobacillus murinus* bacterial Rib_gene0115 **(c)**, Rib_gene1850 **(d)**, Rib_gene2116 **(e)** and Rib_gene2117 **(f)** with GV-971. For static binding, we showed the docking graph (left) and connection types of stable docking (right) between GV-971 and protein. Hydrogen bonds and salt bridges are depicted as yellow and purple dashed lines, respectively. For molecular dynamics, we showed stable binding graph (left), root mean square deviation (RMSD) values (middle), and the distance between ligand and protein (right) of GV-971 oligomers interacting with each Rib protein using AMBER at total 100 nanoseconds (ns) simulation.

Supplementary Fig. S7: Effect of GV-971 on phenylalanine and isoleucine levels in the co-culture assay.

a-b. The effect of GV-971 of two different doses (200 μg/mL and 400 μg/mL, final concentration) on phenylalanine **(a)** and isoleucine **(b)** release of the 48 hours Tg *L.m.* MRS-HT29 conditioned medium (cond. Medium). a) * *P* = 0.03655, b) * *P* = 0.02458 by One-Way ANOVA.

Supplementary Fig. S1

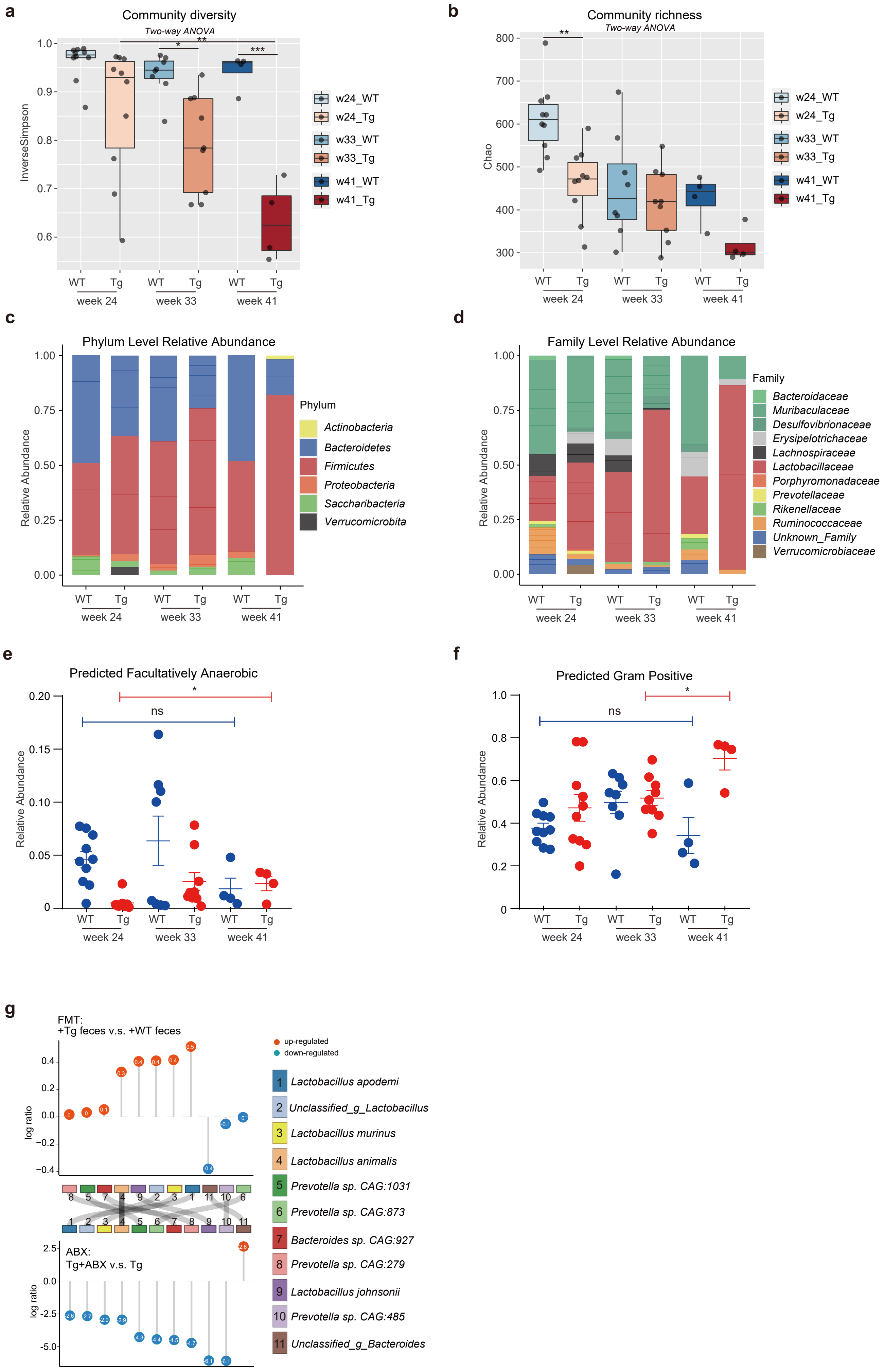

Supplementary Fig. S2

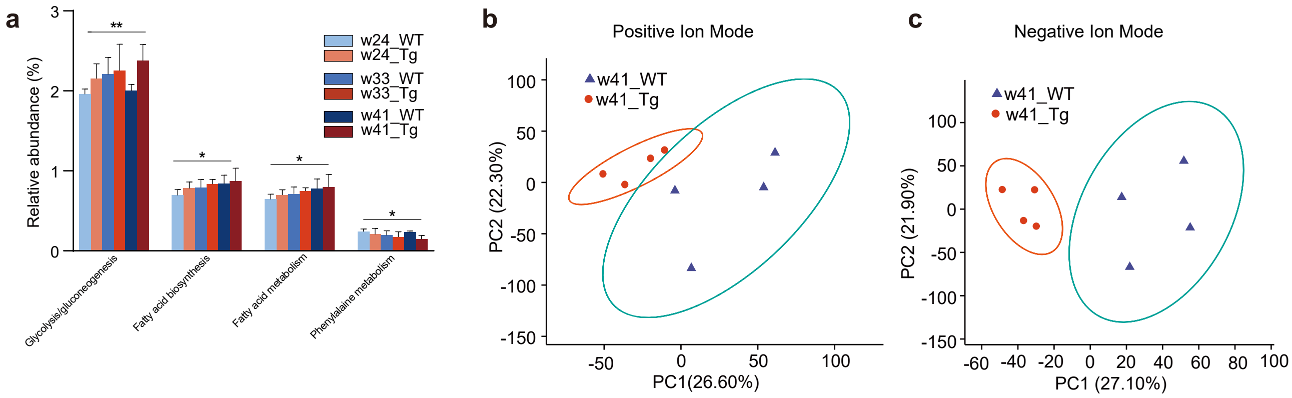

Supplementary Fig. S3

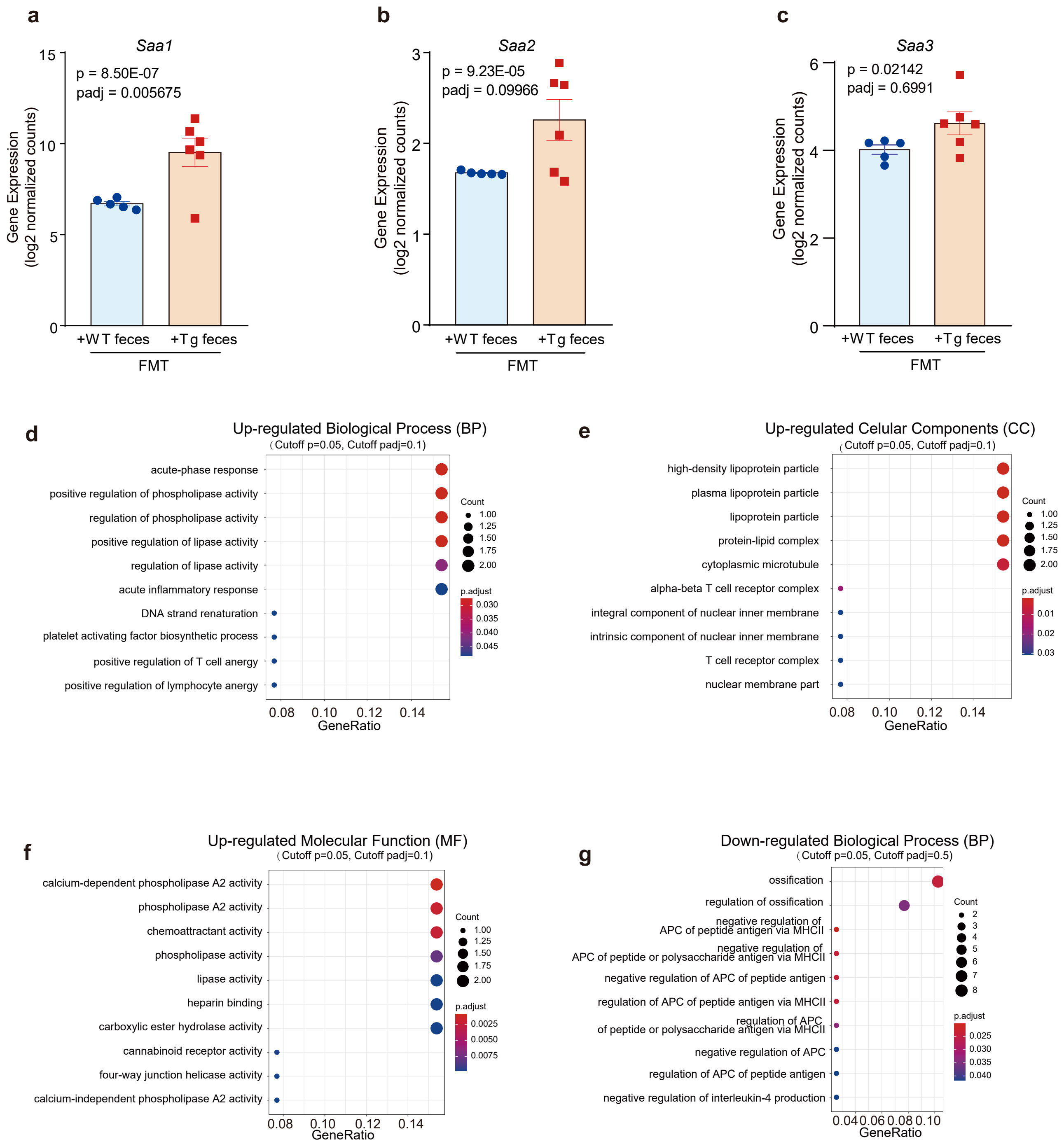

Supplementary Fig. S4

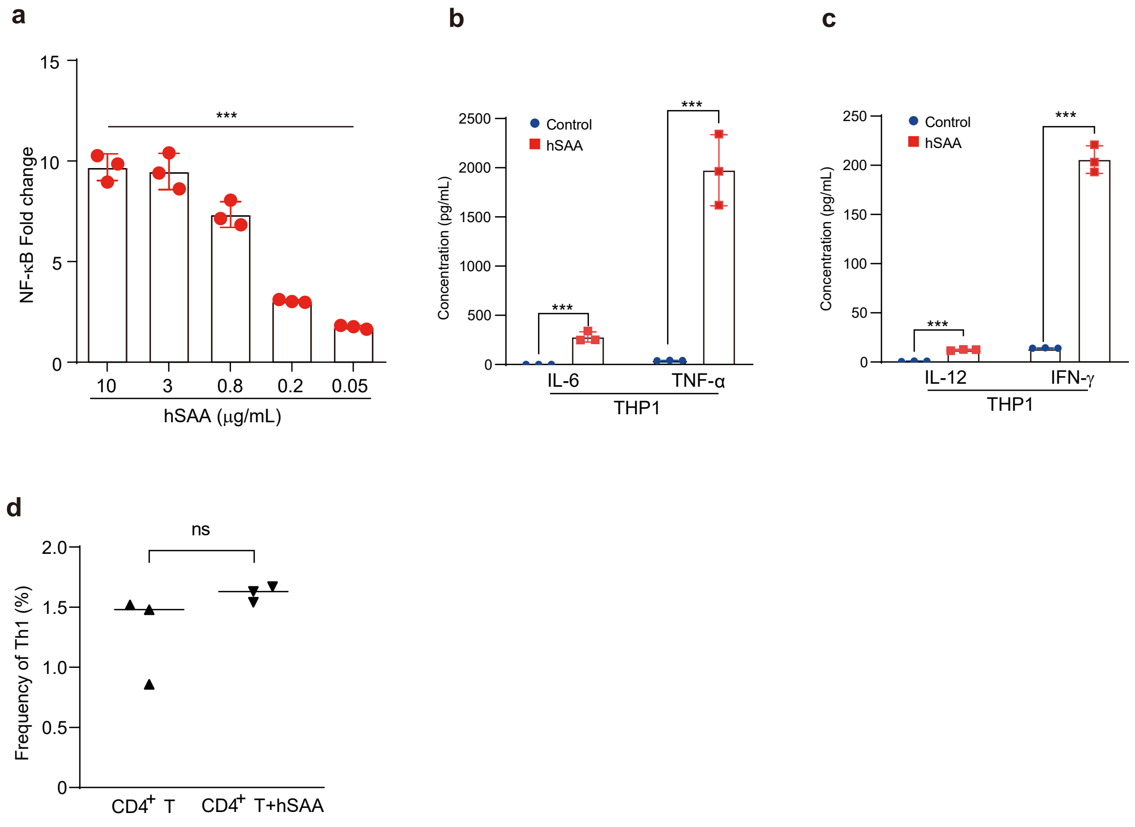

Supplementary Fig. S5

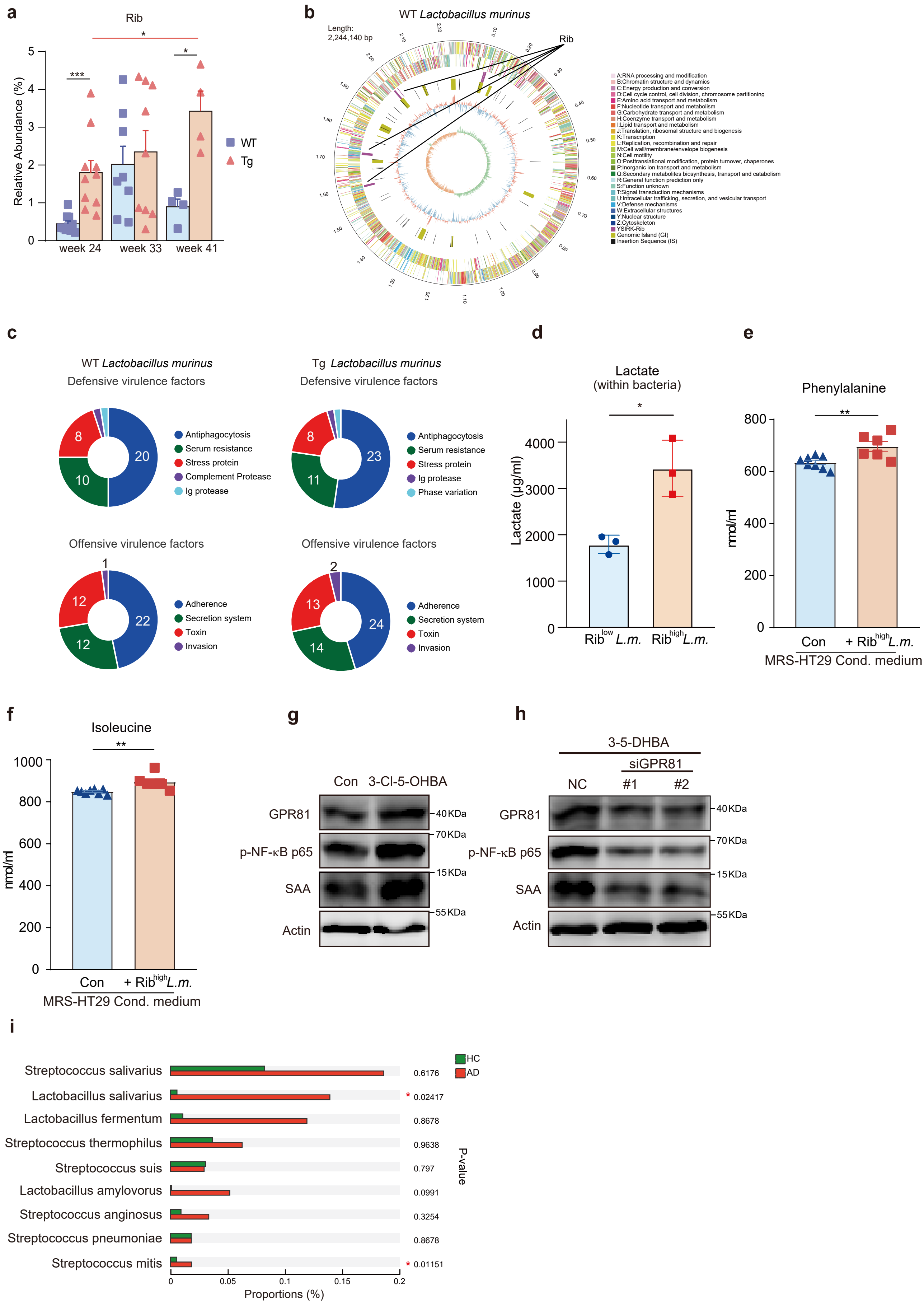

Supplementary Fig. S6

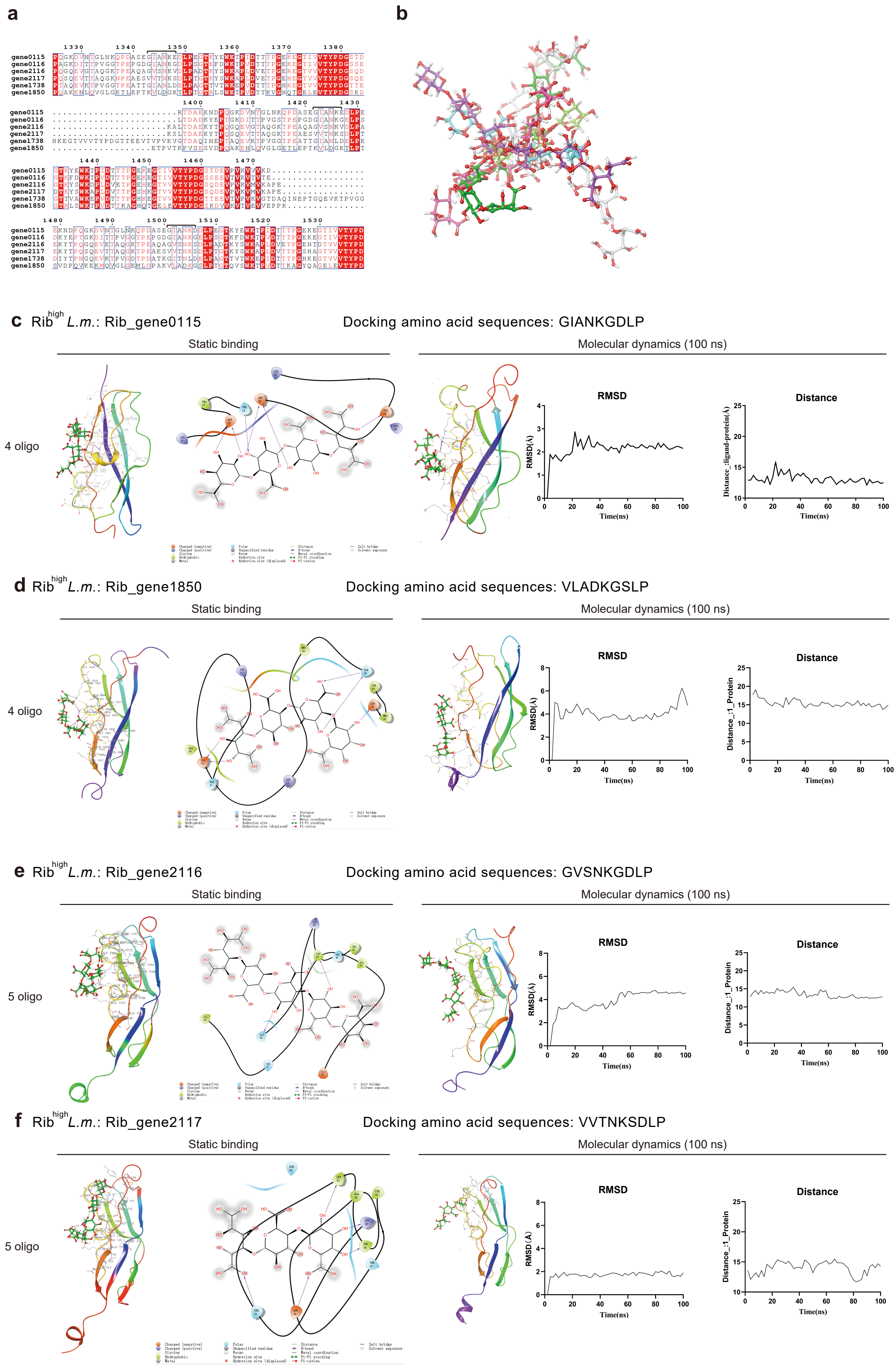

Supplementary Fig. S7

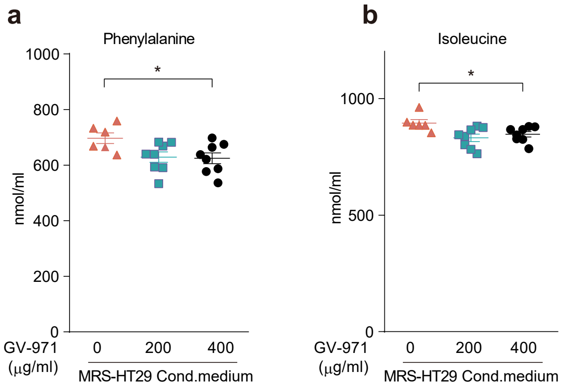

Supplement: Supplementary file 1 — Supplementary Information [file 41421_2024_725_MOESM1_ESM.pdf]
